# Keratinocyte Autophagy‐Mediated Self‐Assembling Tetrahedral Framework Nucleic Acid Induces Wound Healing and Reduces Scar Hyperplasia

**DOI:** 10.1002/mco2.70355

**Published:** 2025-09-16

**Authors:** Jian Jin, Jia‐Jie Li, Zi‐Han Tao, Rong‐Jia Li, Zi‐Liang Zhang, Qing‐Song Liu, Zheng‐Li Chen, Ji‐Qiu Chen, Chen‐Ru Wei, Lei Liu, Liang‐Liang Zhu, Shi‐Hui Zhu, Yun‐Feng Lin

**Affiliations:** ^1^ State Key Laboratory of Molecular Engineering of Polymers Department of Macromolecular Science Fudan University Shanghai China; ^2^ Shanghai Depeac Biotechnology Co., Ltd Shanghai China; ^3^ Department of Burn and Plastic Shanghai Children's Medical Center affiliated to Shanghai Jiao Tong University School of Medicine Shanghai China; ^4^ State Key Laboratory of Oral Diseases National Clinical Research Center for Oral Diseases West China Hospital of Stomatology Sichuan University Chengdu China; ^5^ Department of Burn Surgery The First Affiliated Hospital of Naval Medical University Shanghai China

**Keywords:** autophagy, mTOR/AMPK/ULK 1, scar, tetrahedral framework nucleic acid, wound healing

## Abstract

Tetrahedral framework nucleic acid (tFNA) efficiently treats various diseases; however, its effect on wound healing is unknown. We investigated tFNA's impact on human immortalized epidermal cells (HaCaT) cells and wound healing through in vitro and in vivo experiments. The tFNA is taken up by cells and exhibits good biocompatibility. Transmission electron microscopy and autophagic flux assays showed that tFNA substantially increased the number of intracellular autophagosomes, thus suggesting the activation of cell autophagy. Immunofluorescence and western blotting results indicated decreased microtubule‐associated protein 1 light chain 3I (LC 3I) and prostacyclin (P62) levels, and increased microtubule‐associated protein 1 light chain 3II (LC 3II), suggesting increased autophagic activity. Adenosine 5′‐monophosphate‐activated protein kinase (AMPK) and unc‐51‐like kinase 1 (ULK1) activation, and mechanistic target of rapamycin (mTOR) inhibition were also observed, suggesting their involvement in tFNA‐induced cell activation. Autophagy‐related protein (*ATG*) 5 and *ATG7* knockdown in HaCaT cells reverse confirmed these results. Animal experiment results mirrored the cellular findings, revealing autophagy induction, wound healing promotion, and effective scar score reduction. These results suggest that tFNA promotes HaCaT cell autophagy activation through mTOR pathway inhibition, promoting wound healing and reducing scarring. Our findings expand the application of tFNA and highlight new avenues for clinical wound treatment.

## Graphical abstract

The tFNA consists of four single‐stranded DNAs. The tFNA is taken up by HaCaT cells, activates AMPK, inhibits mTOR, phosphorylates the Ser555 site of ULK1, and thus activates cellular autophagy. After HaCaT autophagy is activated, cell proliferation and migration activity are enhanced, promoting wound healing, reducing scar hyperplasia, and even inducing scar‐free healing.

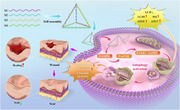

## 1 Introduction

Trauma often leads to the appearance of wounds and scar hyperplasia during the healing process [1]. The appearance of wounds leads to the disappearance of the skin barrier, causing an imbalance in the internal environment of the body, and in severe cases, it can lead to death [2]. In addition, scar hyperplasia may also occur during the wound healing process. Scar formation occurs, especially poor epithelialization, can cause excessive proliferation of fibroblasts, and the deposition of collagen can indirectly lead to the appearance of hypertrophic scars. These scars can have long‐lasting effects on local appearance and function, significantly impacting patients, their families, and society. Another consideration is that cicatricial carcinoma can threaten a patient's life [3, 4].

Epithelialization, a key step in wound healing, directly influences healing and its quality. Insufficient epithelialization can lead to recurrent wound dehiscence and extend the healing cycle, while delayed epithelialization leads to excessive proliferation of other cells (such as fibroblasts) in the wound as well as collagen deposition, resulting in scar hyperplasia. However, the regulation of epithelialization makes healing difficult. Appropriate epithelialization is necessary in order to induce wound healing and improve the healing quality. Excessive epithelialization is associated with hypertrophic scarring and other skin scarring disorders. Delayed or insufficient epithelialization is a key factor in chronic wound development [5, 6, 7]. Appropriate epithelialization not only provides a good barrier for the wound but also enables in situ regeneration of wound tissue, inducing a regenerative wound‐healing pattern. Keratinocytes are the key cell for epithelialization, and often the first to be damaged in wounds. Activation of these cells aids in the healing process [8, 9]. Therefore, they have become important targets for wound repair research.

The tFNA is a DNA nanomaterial based on a tetrahedral structure composed of four DNA strands. It demonstrates excellent biocompatibility and stability, making it suitable for clinical applications [10]. Previous studies have suggested that tFNA mediates multiple biological activities, such as promoting cell proliferation and migration, and it has been shown to be therapeutically effective in studies of trauma, wound, and inflammatory diseases. Recent studies on the mechanism of action of tFNA suggest that autophagy is an important factor, providing a series of activities such as preventing bone necrosis and delaying skin aging [11, 12, 13].

Autophagy is associated with the outcome of many diseases and is an important pathophysiological cellular activity. Autophagy is involved in the entire wound healing process and is associated with scar hyperplasia. Appropriate autophagy can promote healing and reduce scar hyperplasia [14]. Previous research, including our own latest study [15], has suggested that trauma itself will activate wound cell autophagy, but the degree of activation is not sufficient to cope with the damage caused by trauma, and if external intervention can further induce cellular autophagy, it will be beneficial to promote wound healing and reduce scar hyperplasia. Autophagy is an important pathway in wound healing, particularly in the activation of keratinocytes [15].

Given the recent evidence that establishes the role of tFNA in autophagy and the latest research indicating autophagy's involvement in wound healing, the logical next step is to explore the impact of tFNA on wound healing. Therefore, in the present study, we investigated the effects of tFNA on wound‐healing and scar reduction in full‐thickness skin defects through a combination of in vitro cellular experiments and in vivo animal studies. By understanding how tFNA activates autophagy in keratinocytes, our study has aimed to expand the clinical applications of tFNA and provide new possibilities for wound treatment.

## 2 Results

### 2.1 Characterization of tFNAs

**FIGURE 1:**
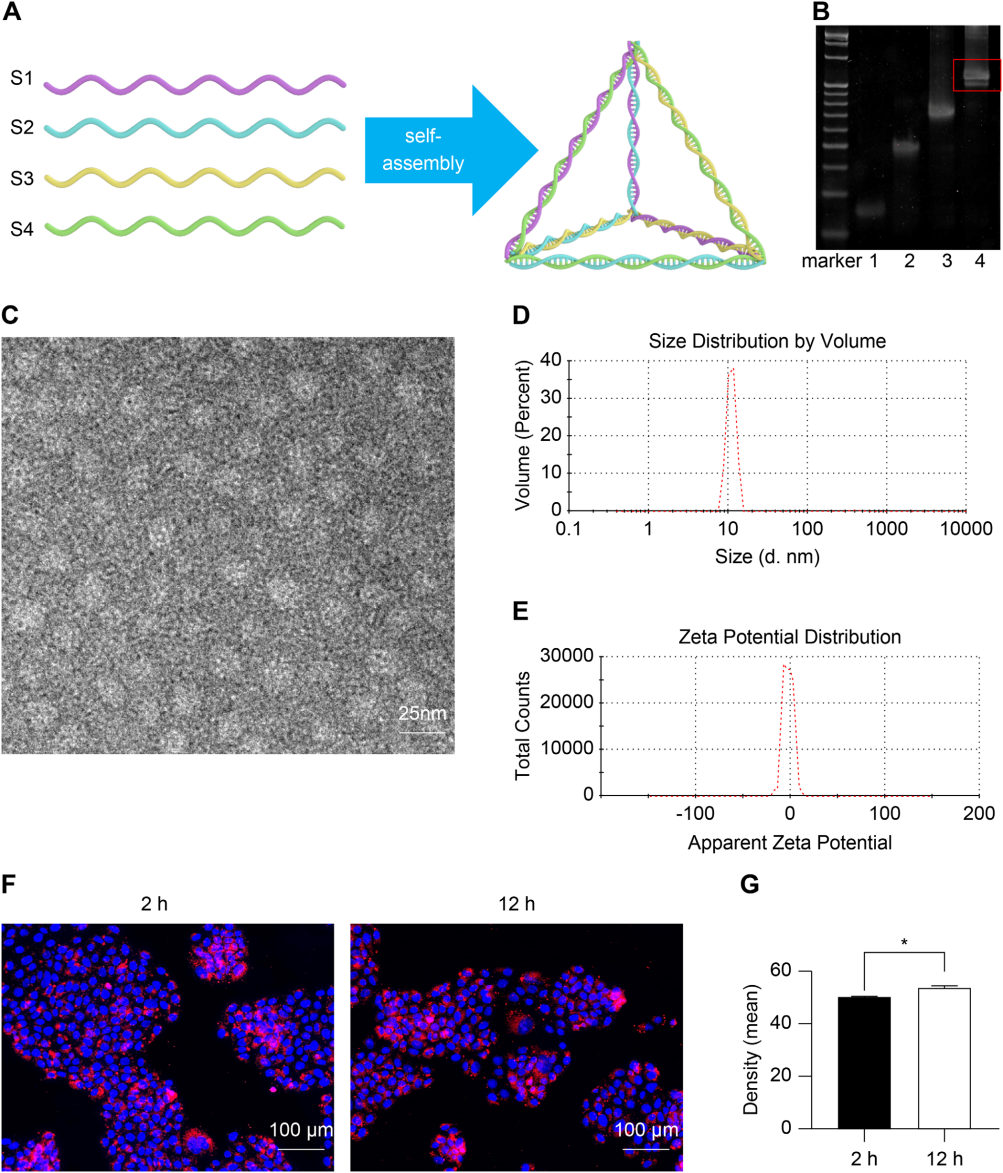
Characteristics of tFNAs. (A) Schematic diagram of tFNA synthesis. (B) Polyacrylamide gel electrophoresis confirming the synthesis of tFNA. Lane 1, S1; lane 2, S1+S2; lane 3, S1+S2+S3; lane 4, S1 + S2 + S3 + S4 (tFNA). (C) Scanning electron microscopy image of tFNA, showing a diameter of approximately 10 nm. (D) Dynamic light scattering showing the tFNA diameter to be approximately 10 nm. (E) ζ Potential indicating the tFNA potential to be −2.61 mV. (F and G) Immunofluorescence staining (F, red fluorescence light) and quantification (G) confirming tFNA uptake by HaCaT, *n* = 5. *p < 0.05. HaCaT, human immortalized epidermal cells; tFNA, tetrahedral framework nucleic acid.

tFNA consists of four ssDNA molecules that form a tetrahedron based on complementary base pairing (Figure 1A). Polyacrylamide gel electrophoresis revealed that ssDNAs were used to synthesize the tFNA and confirmed the molecular weight of the tFNA (Figure 1B), which indicated the successful synthesis of the tFNA. The transmission electron microscope (TEM) results indicated the presence of a 10‐nm‐sized tetrahedra, consistent with the size of tFNA determined through dynamic light scattering (Figure 1C,D). The ζ potential assay indicated a negative potential (−2.61 mV; Figure 1E).

### 2.2 tFNA Concentration Screening

The proliferation and migration of HaCaT cells were correlated with tFNA concentrations. The effects of co‐culturing HaCaT cells with different concentrations of tFNA on proliferation and migration were determined through cell counting kit‐8 (CCK8) (Figure S1A), 5‐ethynyl‐2'‐deoxyuridine (EdU) (Figure S1B,C), and scratch assays (Figure S1D,E). At lower concentrations, their proliferation and migration were enhanced with increasing concentration; however, above 100 nM, increased tFNA concentrations did not further increase but instead decreased HaCaT cell proliferation and migration.

### 2.3 Cellular Uptake of tFNA

Immunofluorescence staining confirmed that tFNA was taken up by HaCaT cells (Figure 1F,G). Flow cytometry analysis showed that 98.50 ± 0.22% and 99.61 ± 0.07% of cells were cyanine 5 (Cy5) positive for 2 and 12 h, respectively. The uptake rate at 12 h was significantly higher than that at 2 h, indicating that HaCaT cells were continuously consuming tFNA. In addition, the uptake rate of HaCaT was significantly higher than that of other wound cells, such as human skin fibroblasts (BJ) and human microvascular endothelial cells (HMEC) (*p* < 0.05) (Figure S2).

### 2.4 Biocompatibility of 100 nM tFNA

At the concentration of 100 nM, the cell morphology did not show significant deformation according to cytotoxicity testing (Figure S3A), but they grew well. Cell viability was 112.38 ± 10.88% and cytotoxicity was 0. The erythema and edema scores were zero in the experimental group during the intracutaneous irritation testing at 24 h (Figure S3B). At 24 and 48 h, no erythema and edema were observed in the experimental groups, so tFNA has no allergenicity (Figure S3C). The pyrogenicity testing results indicated that the temperatures of rabbits increased by 0.40°C, 0.40°C, and 0.35°C, respectively, signaling no pyrogenicity.

### 2.5 tFNA Induces Cellular Autophagy by mTOR/AMPK/ULK 1 in Cell Experiments

#### 2.5.1 Autophagy

**FIGURE 2:**
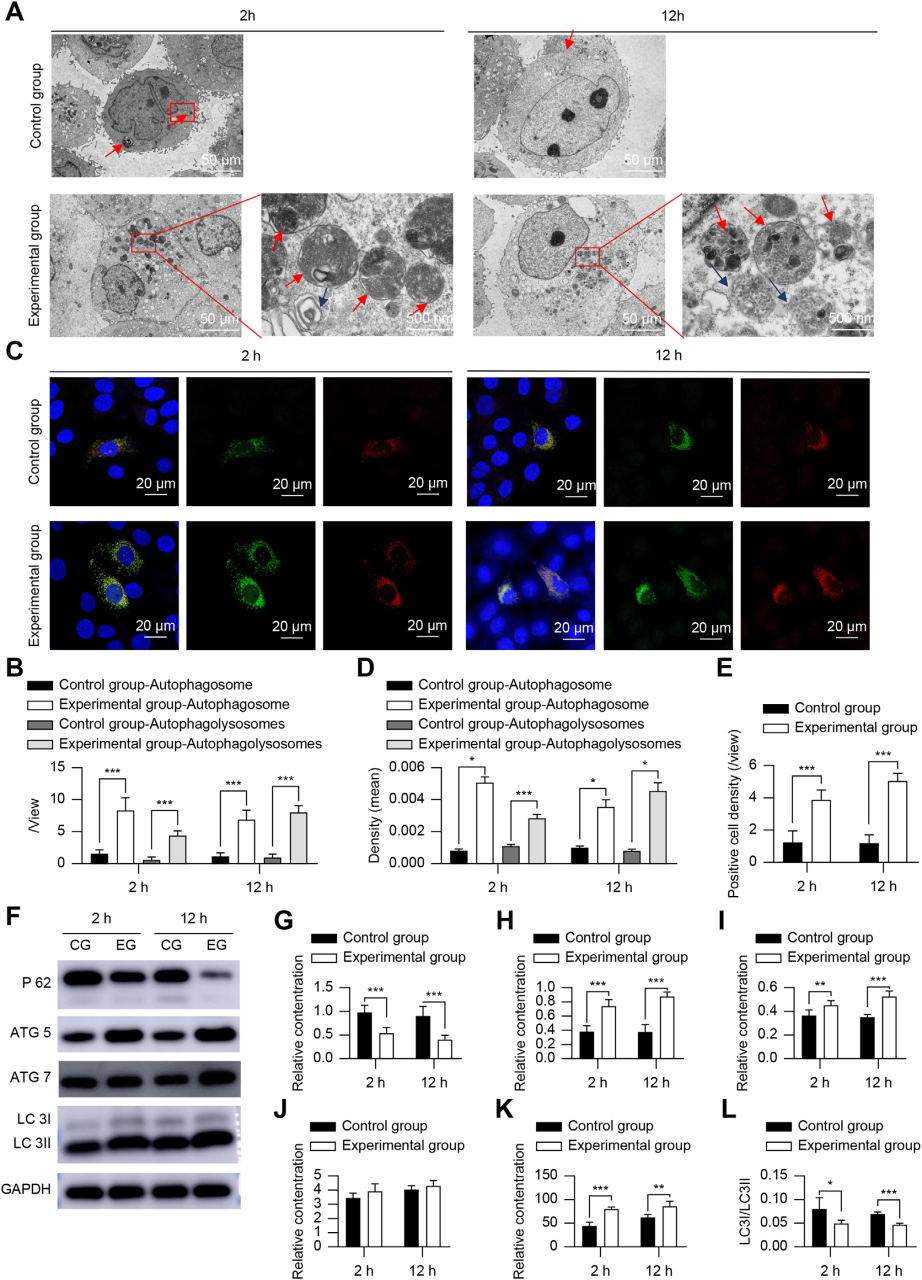
tFNA‐induced HaCaT autophagy. (A and B) Transmission electron microscopy images showing autophagosomes (red arrows) and autophagolysosomes (blue arrows) (A) and their quantification (B). (C–E) Immunofluorescence staining showing autophagic flux (C, green fluorescence indicates autophagosomes, red fluorescence indicates microautophagosomes) and its quantification (D, E). (F–L) Western blot analysis (F) and quantification of the autophagy‐related proteins P62 (G), ATG5 (H), ATG7 (I), LC3I (J), LC3II (K), and LC3I/LC3II (L) levels. *n* = 5. *p < 0.05, **p < 0.01, ***p < 0.001. ATG5, autophagy‐related protein 5; ATG7, autophagy‐related protein 7; GAPDH, glyceraldehyde 3‐phosphate dehydrogenase; HaCaT, human immortalized epidermal cells; LC3I, low complexity communications codec I; LC3II, low complexity communications codec II; P62, ubiquitin‐binding protein p62; tFNA, tetrahedral framework nucleic acid.

TEM observations showed that autophagic activity was significantly enhanced in the experimental group. More autophagosomes and autophagolysosomes were observed, and their numbers were increased after 2 h of co‐culture. Furthermore, the increase in the number of autophagolysosomes became even more pronounced at 12 h (Figure 2A,B).

In autophagic flux assays, autophagosomes appeared green and autophagolysosomes appeared red, and the results were consistent with those obtained from the TEM. The fluorescence intensity corresponding to both autophagosomes and autophagolysosomes was significantly increased at 2 h, and that of autophagolysosomes was even more pronounced at 12 h (*p* < 0.05) (Figure 2C,D). The number of cells undergoing autophagy in the experimental group was also significantly increased (*p* < 0.05) (Figure 2E).

**FIGURE 3:**
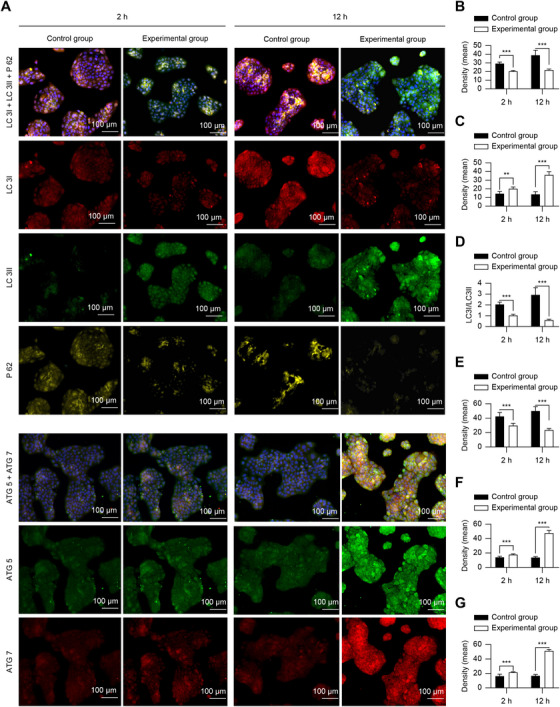
tFNA‐induced autophagy‐related protein expression in HaCaT. (A–G) Immunofluorescence staining (A) and quantification of LC3I (B), LC3II (C), LC3I/LC3II (D), P62 (E), ATG5 (F), and ATG7 (G) expression. *n* = 5. *p < 0.05, **p < 0.01, ***p < 0.001. ATG5, autophagy‐related protein 5; ATG7, autophagy‐related protein 7; HaCaT, human immortalized epidermal cells; LC3I, low complexity communications codec I; LC3II, low complexity communications codec II; P62, ubiquitin‐binding protein p62; tFNA, tetrahedral framework nucleic acid.

Immunofluorescence showed that the LC3I fluorescence intensity was decreased, LC3II fluorescence intensity was increased, the LC3I/LC3II ratio was decreased, the fluorescence intensity of ATG5 and ATG7 was increased, and the fluorescence intensity of P62 was decreased, particularly at 12 h when it was most pronounced (*p* < 0.05) (Figure 3A–G). Western blotting results were consistent with immunofluorescence results. Except for LC3I, there was no significant difference between the control group and the experimental group (*p >* 0.05) (Figure 2F–L).

#### 2.5.2 Other Biological Activity

The intensity of caspase‐3 immunofluorescence and the number of high mobility group box 1 protein (HMGB1) extracellular positive cells were significantly reduced in the experimental group compared to that in the control group (*p* < 0.05) (Figure S4).

#### 2.5.3 Autophagy Signaling Pathways

**FIGURE 4:**
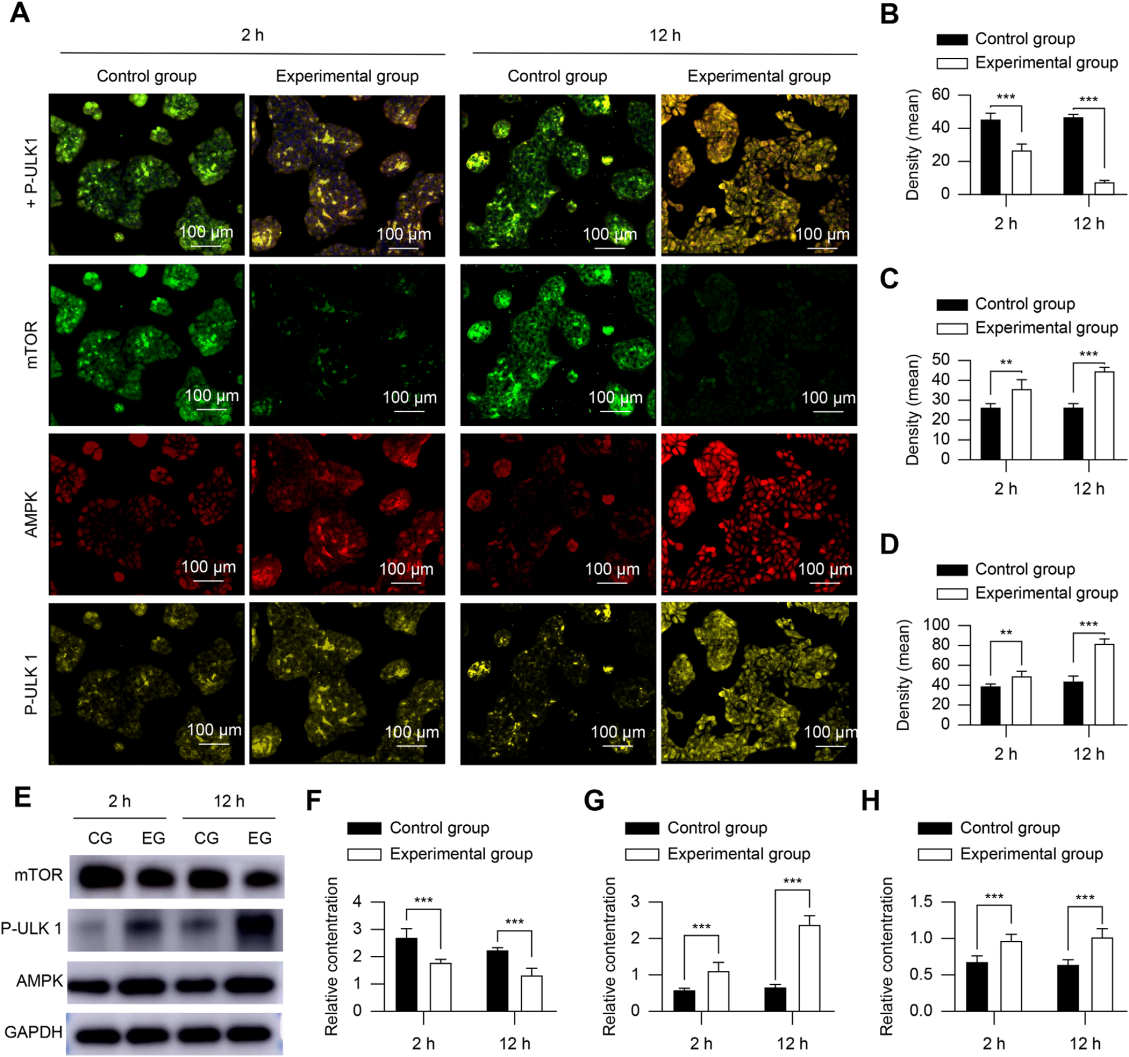
tFNA‐induced autophagy‐related signaling pathways in HaCaT. (A–D) Immunofluorescence staining (A) and quantification of mTOR (B), AMPK (C), and p‐ULK1 (D) expression. (E–H) Western blot analysis (E) and quantification of mTOR (F), AMPK (G), and p‐ULK1 (H) levels (CG, control group; EG, experimental group). *n* = 5. *p < 0.05, **p < 0.01, ***p < 0.001. AMPK, adenosine 5′‐monophosphate‐activated protein kinase; GAPDH, glyceraldehyde‐3‐phosphate dehydrogenase; HaCaT, human immortalized epidermal cells; mTOR, mammalian target of rapamycin; p‐ULK1, phosphorylated Unc‐51‐like autophagy protein 1; tFNA, tetrahedral framework nucleic acid.

Immunofluorescence results showed decreased mTOR fluorescence intensity and enhanced AMPK and p‐ULK1 (Ser555) fluorescence intensity (*p* < 0.05). The western blotting results were consistent with immunofluorescence results (Figure 4).

#### 2.5.4 Gene Knockdown Reverse Validation

Gene knockdown information and human ATG5 and ATG7 knockout in HaCaT information is detailed in Supporting Information. After the double knockdown of *ATG5* and *ATG7*, immunofluorescence results showed decreased mTOR fluorescence intensity and enhanced AMPK and p‐ULK1 (Ser555) fluorescence intensity (*p* < 0.05), but no significant changes in the fluorescence intensities of LC3I, LC3II, LC3I/LC3II, P62, HMGB1, and caspase‐3 (*p* > 0.05) (Figure S5). In addition, CCK8 showed no significant difference in cell proliferation (97.41 ± 4.19% vs. 97.01 ± 4.57, *p* > 0.05), and the scratch assay showed no significant difference in cell migration (24.64 ± 2.62% vs. 25.70 ± 3.40, *p* > 0.05).

### 2.6 tFNA Promotes Wound Healing and Reduces Scar Hyperplasia by Inducing Cellular Autophagy in Animal Experiments

Wound healing was correlated with tFNA concentration, and the results were generally consistent with those of the cellular experiments. Administration of 100 nM tFNA yielded better wound‐healing and scar improvement results, with higher rates of wound healing and shorter healing times at all stages (Figure S6).

**FIGURE 5:**
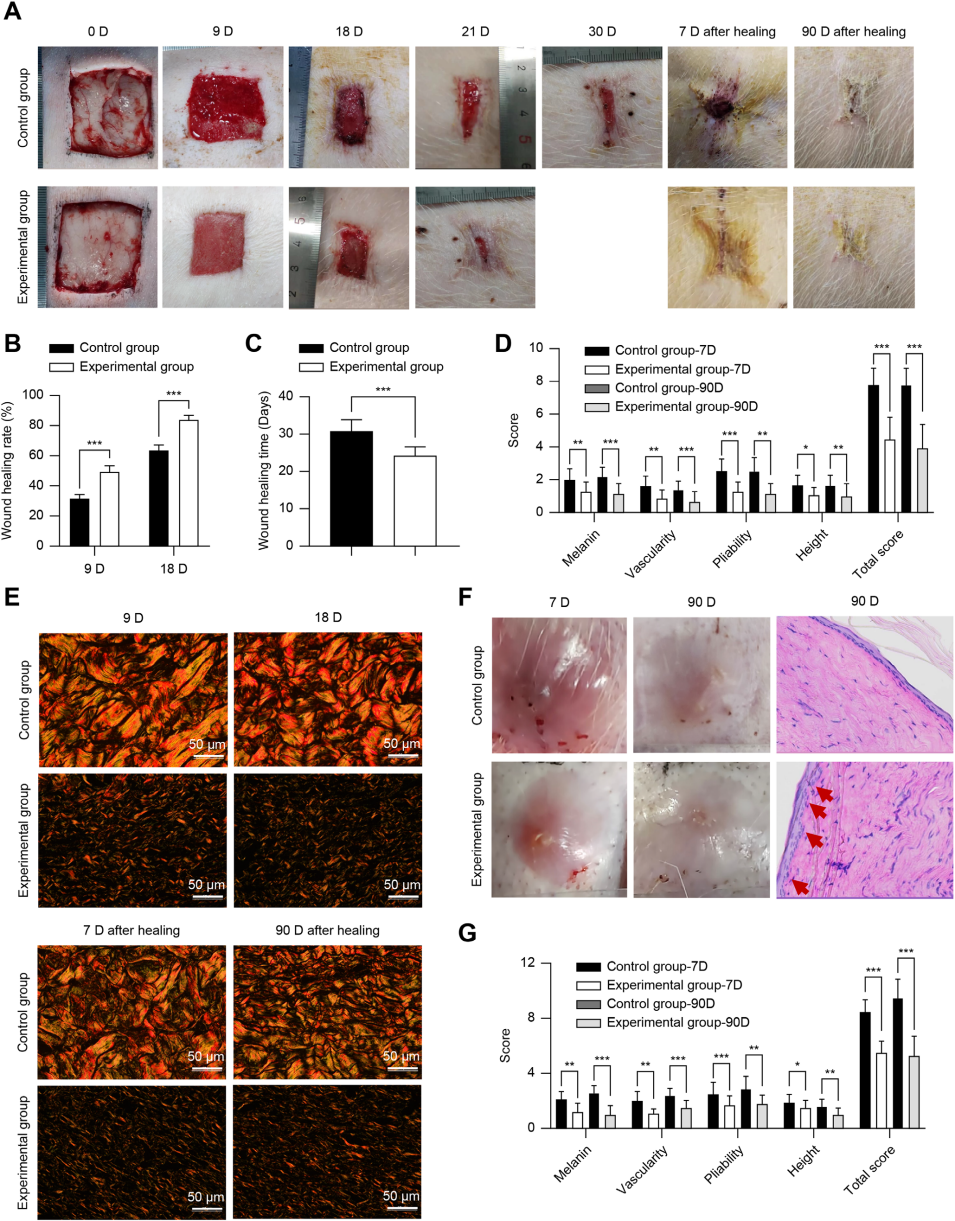
Wound healing and scar hyperplasia in an animal model. (A–C) Photographs depicting wound healing (A) and quantification of the healing rate (B) and healing time (C). (D) VSS of scars after wound healing in Bama miniature pigs. (E) Sirius red represents picric acid staining, yellow polarized light represents type I collagen, green polarized light represents type III collagen. (F and G) Photographs, HE staining (F), and VSS (G) of rabbit ear scars. Red arrows indicate skin ridge formation. *n* = 15. *p < 0.05, **p < 0.01, ***p < 0.001. VSS, Vancouver scar scale.

At a local injection concentration of 100 nM, the wound‐healing rate of the experimental group was higher than that of the control group at 9 D and 18D (Figure 5A,B), and the healing time was shorter than that of the control group (*p* < 0.05) (Figure 5C). Individual and total VSS scores were lower than those of the control group (*p* < 0.05) (Figure 5D). Sirius red picric acid staining (SR‐PA) staining, the deposition of type I collagen in the control group was observed, and the collagen arrangement was disordered. The experimental group showed an increase in type III collagen and a slightly more orderly arrangement of collagen, indicating an improvement in scar hyperplasia. Additionally, no significant deposition of type I collagen was observed, indicating that it is not pathological scar hyperplasia (Figure 5E). The scar score of the rabbit ear scars was the same, and the long‐term anti‐scar effect was significant (*p* < 0.05) (Figure 5F,G). HE staining revealed that some scars were non‐pathological, with skin ridge structures.

**FIGURE 6:**
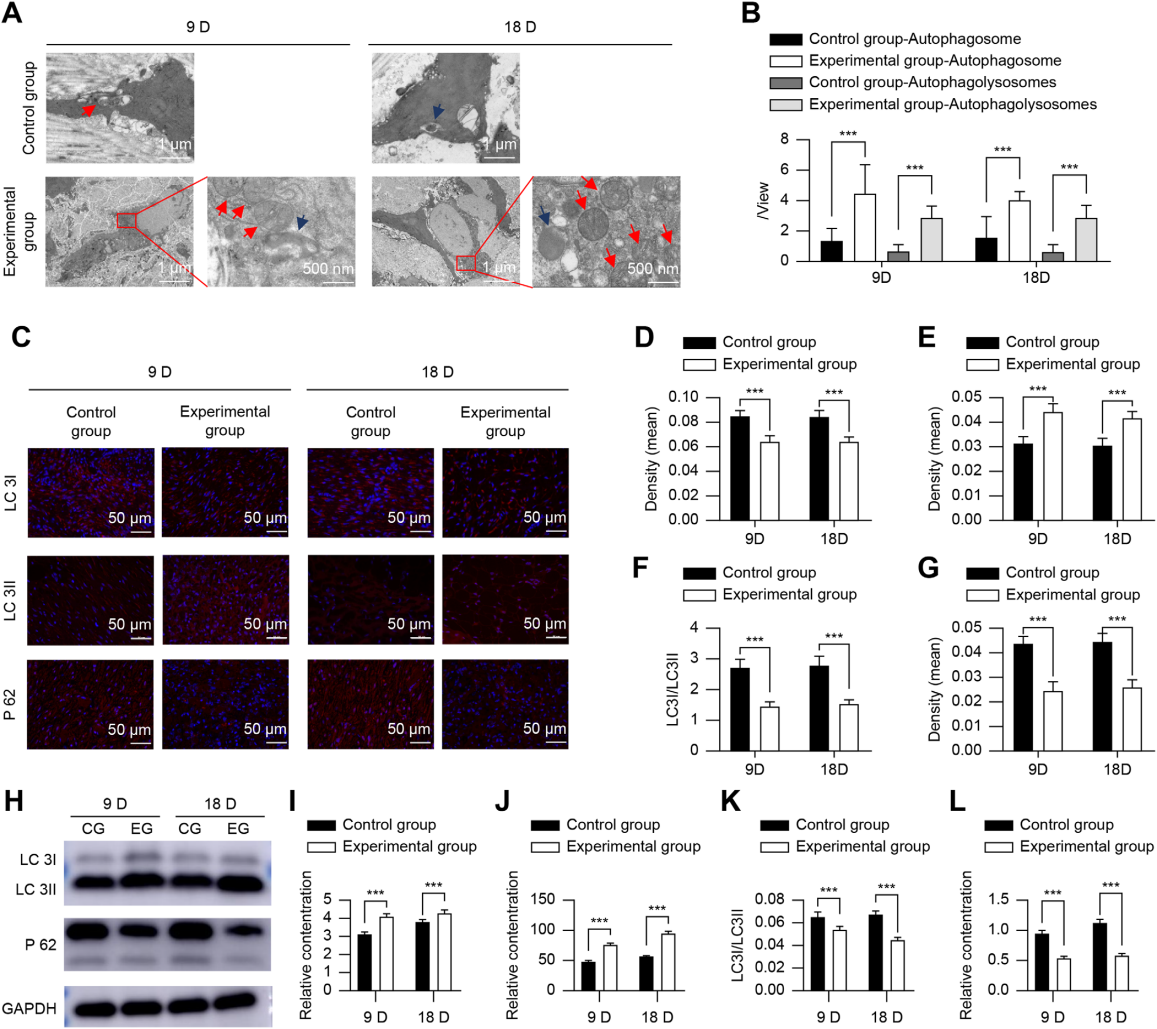
Cell autophagy in wound tissue. (A and B) Transmission electron microscopy images showing autophagosomes (red arrows) and autophagolysosomes (blue arrows) (A) and their quantification (B). (C–G) Immunofluorescence staining (C) and quantification of the autophagy‐related proteins LC3I (D), LC3II (E), LC3I/LC3II (F), and P62 (G) expression. (H–L) Western blot analysis (H) and quantification of the autophagy‐related proteins LC3I (I), LC3II (J), LC3I/LC3II (K), and P62 (L) levels. *n* = 15. *p < 0.05, **p < 0.01, ***p < 0.001. GAPDH, glyceraldehyde‐3‐phosphate dehydrogen; LC3I, low complexity communications codec I; LC3II, low complexity communications codec II; P62, ubiquitin‐binding protein p62.

The TEM results indicated more autophagosomes and autophagolysosomes in the experimental group than in control group (Figure 6A,B). Immunofluorescence indicated decreased fluorescence intensity of LC3I and P62, increased fluorescence intensity of LC3II, ATG5, and ATG7, and decreased LC3I/LC3II ratio compared to those in the control group (*p* < 0.05) (Figure 6C–G). For western blotting, except for the content of LC3I, which is opposite to immunofluorescence, other indicators are consistent with immunofluorescence, but do not affect LC3I/LC3II (Figure 6H–L).

The experimental group exhibited a significant decrease in HMGB1 and caspase‐3 immunofluorescence and a significant increase in the number of cells positive for CD31 immunofluorescence (*p* < 0.05) (Figure S7).

## 3 Discussion

Traumatic injuries often result in post‐healing scar formation, which can lead to longer lasting and serious effects [16]. Autophagy, a protective mechanism activated in response to complex environments, plays an important role in wound healing—from initial wounding to healing and even to scar formation. tFNA, a DNA‐based material, can be taken up through the cell membrane and produces various pathophysiological effects in the cell, among which a notable function is autophagy activation [17, 18]. In the present study, we investigated the role of tFNA in activating autophagy in the context of wound healing and scar formation. We synthesized tFNA with a stable spatial structure from four ssDNAs. Successful synthesis and spatial structure and size were observed using TEM. The results confirmed the ability of tFNA to enter the cell via the cell membrane and exert its biological effects. In addition, tFNA enhanced membrane‐crossing ability compared to that of its constituent ssDNAs, suggesting the advantageous role of its spatial structure. Subsequently, cell experiments and animal experiments verified that it activates autophagy, further promoting wound healing, reducing scar hyperplasia, and even inducing scarless hyperplasia.

The impact of tFNA on cells showed a concentration‐dependent trend, which is consistent with previous studies [10, 13], with the most effective outcome at a concentration of 100 nM in HaCaT cells. This concentration yielded consistent results in cellular experiments and animal models, including cell proliferation, migration, wound healing, and scar formation assays. Therefore, all subsequent experiments were conducted at this concentration. Although HaCaT is not the only cell in the wound that can take up tFNA, its uptake rate is significantly higher than that in other cells, and almost all cells take up tFNA. Moreover, tFNA exhibited good biocompatibility at this level, meeting cytotoxicity, skin irritation, allergenicity, and pyrogenicity requirements for potential clinical applications as per relevant ISO standards [19, 20].

Increased autophagic activity in HaCaT cells was directly observed under a TEM, revealing the prevalence of autophagosomes at early stages, with autophagolysosome content increasing significantly over time. Autophagolysosomes are formed by the combination of autophagosomes and lysosomes. They are the key link of organelle degradation and reuse after autophagy activation. They appear later than autophagosomes, which is the manifestation of autophagy being fully activated [21]. TEM also suggests that even without the action of tFNA, autophagy occurs in cells, but the autophagy activity is low, which means that the basic level of autophagy is not sufficient to cope with cell damage, and external intervention is therefore needed to further activate autophagy to produce sufficient cell protection.

Autophagic flux assays further validated tFNA‐activated autophagy completely in HaCaT cells. In addition, co‐culturing HaCaT cells with tFNA led to the degradation of LC3I to LC3II, resulting in a lower LC3I/LC3II ratio, which is also a typical manifestation of autophagy being activated [14, 15]. This corresponded to increased levels of the autophagy‐associated proteins ATG5 and ATG7, and a significant decrease in P62, which showed immunofluorescence co‐localization with LC3, confirming increased levels of autophagy [22]. The mTOR pathway plays an important role in autophagy activation [21]. Co‐culturing HaCaT cells with tFNA led to increased AMPK levels, whereas inhibiting the mTOR pathway resulted in a significant decrease in mTOR levels, subsequently increasing ULK1 (Ser555) levels. This suppressed the inhibitory effect of the mTOR pathway on autophagy, resulting in increased autophagic activity. This signaling pathway acts prior to the activation of autophagy by the ATG proteins. Reverse genetics validation was performed using HaCaT cells with knockdown of the *ATG5* and *ATG7* genes. Despite tFNA affecting the AMPK‐mTOR‐ULK1 (Ser555) axis in these cells, it was unable to activate autophagy, suggesting that tFNA activates autophagy in HaCaT cells by triggering AMPK, inhibiting mTOR, thereby increasing ULK1 phosphorylation at Ser555, and weakening its inhibition on ATG genes. Although mTOR, an important signaling pathway for cell proliferation, showed reduced activity in this study, it contradicted the results of enhanced cell proliferation activity. This may be because mTOR signaling is not the only pathway that promotes cell proliferation, and tFNA may lead to proliferation through other signaling pathways. In addition, damage to the control group cells did not lead to cell death or inactivation. However, the protective effect of tFNA in the experimental group reduced the risk of death or inactivation, indirectly providing evidence for cell proliferation.

The Bama miniature pig was used as the model organism for our animal experiments owing to its skin structure, which more closely resembles human skin compared to the commonly used rodent models. It provides ample space for preparing an autologously controlled skin defect model [23, 24]. In our study, the skin defect model was created by excising the full thickness of the skin on the back, preventing natural regeneration. TEM results suggested increased autophagic activity in the injured tissue. These results were corroborated through LC3 and P62 immunofluorescence and western blotting. In fact, all cells exhibited LC3II‐positive immunofluorescence, indicating activation of autophagy across all cells. tFNA accelerated wound healing as the rate increased and the healing time decreased. Although Bama miniature pigs do not typically develop pathologic hypertrophic scarring, VSS scores still indicated effective prevention of scar formation. However, the present study primarily focused on assessing the role of tFNA in scar prevention during wound treatment, limiting our ability to evaluate its therapeutic efficacy in managing scar formation. To further validate the efficacy of tFNA in preventing pathologic hypertrophic scarring, we used a rabbit ear scar model, recognized as the gold standard for studying scar formation in animal models. The results confirmed that tFNA prevents pathologic hypertrophic scarring at both early (7 days post‐healing) and late (90 days post‐healing) stages. Some scars even exhibited non‐pathologic hypertrophic scarring and skin ridges, thus suggesting that tFNA has the potential to induce regenerative wound healing.

Both cell and animal experiments have confirmed that tFNA, based on autophagy activation, has the potential to promote wound healing and reduce scar hyperplasia. However, our research also has limitations. Our study focused on an acute wound model, the supporting role for the treatment of chronic wounds is relatively weak. Considering that chronic wounds are of great concern, future studies should validate tFNA's capacity to induce regeneration in chronic wounds. In addition, although the appearance of ridges suggests to some extent regenerative healing and improves skin quality after healing, its relationship with regeneration still needs to be further explored and verified. In animal experiments, we did not conduct targeted exploration on keratinocytes, but focused more on comprehensive effects. The strength of evidence for further verification of autophagy activity in keratinocytes in cell experiments has an impact, and further in‐depth research on target cells is needed in the future. Moreover, a better mechanistic understanding of tFNA's involvement in wound healing is required to establish a robust theoretical basis for its clinical application.

## 4 Conclusion

tFNA effectively crosses the cell membrane and demonstrates biological activity and excellent biocompatibility, making it suitable for clinical applications. In the present study, tFNA promoted HaCaT cell proliferation and migration by activating autophagy. This activation occurred through AMPK activation, leading to the inhibition of the mTOR pathway. The resulting autophagy activation in HaCaT cells not only boosted proliferation and migration but also accelerated wound healing, while reducing scar formation. Furthermore, it facilitated partial regenerative healing to some extent and promoted research on scar‐free healing of wounds.

## 5 Materials and Methods

### 5.1 Reagents

HaCaT, BJ, and HMEC‐1 were purchased from the Shanghai National Cell Bank (Shanghai, China). CCK8 was purchased from Beijing Solaibao Technology Co., Ltd (Beijing, China). Autophagy Assay Kit (ab139484), anti‐LC3A antibody (ab52768), anti‐LC3B antibody (ab243506), anti‐APG5L/ATG5 antibody (ab238092), anti‐ATG7 antibody (ab52472), anti‐SQSTM1/p62 antibody (ab314504), anti‐mTOR antibody (ab32028), anti‐AMPK alpha 1 antibody (ab110036), anti‐ULK1 antibody (ab229537), anti‐HMGB1 antibody (ab79823), and anti‐caspase‐3 antibody (ab184787) were purchased from Abcam (Shanghai) Trading Co., Ltd. (Shanghai, China). BrdU was purchased from Beijing Baiaobo Technology Co., Ltd. (Beijing, China).

### 5.2 Synthesis of tFNA and Physical and Chemical Characterization

tFNA consists of four single‐stranded DNAs (ssDNAs), the details of which are shown in Table 1. Equal amounts of the four ssDNAs were added to TM buffer with 10 nmol L^−1^ Tris‐HCl (pH 8.0) and 50 nmol L^−1^ MgCl_2_ and incubated at 95°C for 10 min to react, after which the mixture was cooled and kept at 4°C for 20 min to react. tFNA synthesis was confirmed using an 8% polyacrylamide gel electrophoresis system (Bio‐Rad, Hercules, CA, USA). The particle size of the molecules was determined using a TEM (FEI Co., Hillsboro, OR, USA) and dynamic light scattering (Malvern Instrument Ltd., Malvern, UK). The ζ potential of tFNA molecules was measured with the Zetasizer Nano ZS90 instrument [25].

**TABLE 1:** Sequences of the four ssDNAs.

ssDNA	Base sequence (5′–3′)
S1	ATTTATCACCCGCCATAGTAGACGTATCACCAGGCAGTTGAGACGAACATTCCTAAGTCTGAA
S2	ACATGCGAGGGTCCAATACCGACGATTACAGCTTGCTACACGATTCAGACTTAGGAATGTTCG
S3	TTGACCTGTGAATTACTACTATGGCGGGTGATAAAACGTGTAGCAAGCTGTAATCGACGGGAAGAGCATGCCCATCC
S4	ACGGTATTGGACCCTCGCATGACTCAACTGCCTGGTGATACGAGGATGGGCATGCTCTTCCCG

Abbreviation: ssDNA, single‐stranded DNA.

### 5.3 Biocompatibility Testing

Biocompatibility test items were selected according to ISO10993 standards, and the test was conducted according to the operation protocols in the corresponding section of ISO10993 [26]. The test items were cytotoxicity, intradermal irritation, sensitization, and heat source, which were tested as follows: (1) Cytotoxicity: The extract was mixed with conventional culture medium at a 1:1 ratio as the culture medium for L929 cells. After 24 h of cultivation, cell proliferation and morphology were observed under a 10× magnification optical microscope (Olympus Co., Tokyo, JPN), and cell toxicity was detected using the CCK8 assay. (2) Intracutaneous stimulation: Extraction solution (1 mL) was injected into the subcutaneous tissue of pre shaved back hair from New Zealand albino rabbits, and skin erythema and lumps were observed after 24 h. (3) Allergenicity: Extraction solution (1 mL) was applied to the skin of guinea pigs with their back hair pre shaved, and redness and swelling of the skin were observed at 24 and 48 h. (4) Thermal source detection: Three New Zealand albino rabbits were injected of tFNA with 10 mL/kg body weight via ear vein, and their body temperature was measured every 30 min for a total of six times. The highest of the six body temperatures was subtracted from the normal body temperature to obtain the temperature increase (°C) of the rabbit. All tests were repeated five times.

### 5.4 Cell Experiments

#### 5.4.1 Determination of Effective Concentration

High‐sensitivity HaCaT cells were co‐cultured with 25, 50, 100, 150, or 200 nM tFNA for 24 h. Cell proliferation was determined using CCK8 and EdU assays. Cell migration was determined using the scratch assay. The optimal effective concentration was evaluated comprehensively based on the changes in cell proliferation and migration. For the CCK8 assay, CCK8 was added, and the cell mix was further cultured for 1 h. The absorbance was measured at a wavelength of 450 nm using a microplate reader (SpectraMax iD5, MOLECULAR DEVICES). Compare the relative proliferation rate of experimental and control groups by absorbance. The relative proliferation rate (%) = (absorbance in the experimental group/control group) × 100% [27]. For EdU staining, BrdU was added and cultured for 2 h. Subsequently, the cells were immobilized, incubated with Apollo staining solution for 30 min, washed, and again incubated with Hirst 33342 staining solution for 30 min. A fluorescence microscope (EVOS M5000, Thermo Fisher Scientific) at 400x magnification was used and randomly five fields of view were selected to calculate the average number of positive cells [28]. For the scratch test, at 0, 12, and 24 h after seeding, one field in each cell well was selected and imaged, and the process was repeated five times. Two lines were drawn to measure the distance between cells based on their migration status. Migration rate (%) = (0‐h distance − measured distance)/(0‐h distance) × 100% [29].

#### 5.4.2 Cellular Uptake

Immunofluorescence staining and flow cytometry were used to assess the uptake of tFNA by HaCaT cells, BJ, and HMEC. HaCaT cells were plated in six‐well plates at a density of 2 × 10^6^ cells/well and co‐cultured with Cy5‐labeled tFNA for 2 h and 12 h. The intensity of Cy5 immunofluorescence indicated the level of intracellular Cy5 content. For the immunofluorescence assay, the cells were immobilized using polyoxymethylene, permeabilized using Triton X‐100, and blocked at 20°C for 1 h. After incubation in the dark with fluorescent secondary antibodies, the counterstained nuclei were mounted with 4',6‐diamidino‐2‐phenylindole. The mean optical density was then calculated [30].

#### 5.4.3 Autophagy and Mechanism of Action

HaCaT cells were co‐cultured with tFNA for 2 and 12 h. The degree of autophagy activation and the stage of autophagy were detected using a TEM (FEI Co.) and an autophagic flux assay kit according to the manufacturer's instructions. The degree of autophagy activation was further assessed by detecting LC3I, LC3II, ATG5, ATG7, and P62 levels through immunofluorescence and western blotting. The signaling pathways activated by autophagy were assessed by determining the changes in mTOR, AMPK, and p‐ULK1 concentrations using immunofluorescence and western blotting. Changes in necrosis and apoptosis were detected using HMGB1 and caspase‐3 immunofluorescence. HMGB1 was assessed using the number of extracellular positive cells, and caspase‐3 was assessed using fluorescence intensity. For bio‐scanning electron microscopy, cells were treated with glutaraldehyde, dried via critical point drying, and then sprayed with metal powder. Autophagosomes were identified and counted, and the average value was calculated. For western blotting analysis, the proteins extracted from the cell samples underwent electrophoresis. They were incubated with primary antibodies, decolorized overnight, incubated with secondary antibodies, and exposed. Then, the gray value was analyzed using ImageJ software (National Institutes of Health, Bethesda, MD, USA) to determine the content. The immunofluorescence assay was performed as described above.

#### 5.4.4 Gene Knockdown Reverse Genetics Validation

For reverse genetics validation, HaCaT cells with knockdown of the *ATG5/ATG7* genes were co‐cultured with tFNA for 12 h. Through immunofluorescence analysis, we then detected LC3I, LC3II, P62, mTOR, AMPK, and p‐ULK1, as well as cell proliferation, migration, caspase‐3, and HMGB1 immunofluorescence in gene‐knockout cells. We then reverse validated that tFNA induces changes in the biological activity of HaCaT cells based on autophagy induction.

### 5.5 Animal Experiments

#### 5.5.1 Model Establishment and Groups

Bama miniature pigs were used as model organisms for skin trauma for determining the local injection concentration of tFNA in animal experiments and for assessing the effect of tFNA on trauma healing. New Zealand White rabbits were used to prepare a rabbit ear scar model to validate the effects of tFNA on scar formation.

Five 3 × 3 cm square full‐thickness skin defects were created on each side of the spine of three pigs. The wounds were injected with 1 mL containing 25, 50, 100, 150, or 200 nM tFNA depending on wound location. The dressing was replaced, and fresh injections were administered every 3 days. The healing rate of the wounds were observed on days 9 and 18, and the healing time was recorded. The most appropriate concentration of tFNA for subsequent experiments was selected based on the healing of these wounds.

Three 3 × 3 cm square full‐thickness skin defects were created on each side of the spine of five pigs. The wounds on the left side were considered the experimental group and the wounds on the right side were considered the control group, resulting in a total of 15 samples in each group. The wound dressings were changed every 3 days. In the control group, the skin was sterilized with chlorhexidine, locally injected with 1 mL normal saline at multiple sites, and sterilized with chlorhexidine again. The wounds were covered with sterile petroleum jelly gauze and sealed with a sterile dressing. The experimental group received local injections of 1 mL tFNA instead of saline, with all other procedures being the same as those of the control group. The wound‐healing rate was observed on days 9 and 18, 30 min after local injection of tFNA. A 1‐mm diameter biopsy instrument was used to collect a full‐thickness wound specimen for electron microscopy, histological examination, and western blotting, and the wound‐healing time was recorded. Wound healing was scored using the Vancouver scar scale (VSS), which is detailed in Supporting Information Methods.

#### 5.5.2 Wound Tissue Autophagy and Other Histological Tests

The degree of autophagy activation was further evaluated through TEM as well as immunofluorescence and western blotting for LC3I, LC3II, and P62. SR‐PA staining evaluates the distribution and content of collagen in scars formed, providing histological evaluation of scars. Apoptosis and necrosis in the traumatized tissue were detected using caspase‐3 and HMGB1 immunofluorescence and expressed as fluorescence intensity. The number of cells positive for CD31 immunofluorescence reflected neovascularization [31]. All procedures were conducted in the same way as the cell experiments.

#### 5.5.3 Rabbit Ear Scars

Five male New Zealand albino rabbits weighing 2750 ± 250 g were used for these experiments. A wound involving the cartilage membrane was made on the ear of a rabbit, with a diameter of 1 cm, and divided into an experimental group and a control group, with 10 samples in each group. A total of 0.1 mL of tFNA was locally injected into each wound in the experimental group, whereas 0.1 mL of normal saline was locally injected in the control group. The dressing was changed once every 3 days, and the healed wounds were left untreated. After 7 and 90 days of wound healing, the scars were assessed using the VSS [4]. On the 90th day, scar tissue was collected and stained with HE.

This study was approved by the Laboratory Animal Management and Use Committee of Huizhi Yinghua Medical Technology R&D (Shanghai) Co., Ltd (approval number: PSH2022‐09192). All animals were purchased from Huizhi Yinghua Medical Technology R&D (Shanghai) Co., Ltd.

### 5.6 Statistical Analyses

Data are expressed as mean ± standard deviation. All data were statistically analyzed using SPSS software version 26.0 (IBM Corp., Armonk, NY, USA). Comparison between two groups were analyzed using the independent samples *t*‐test if the data were normally distributed; otherwise, the Wilcoxon rank sum test was used. Comparison between multiple groups using analysis of variance if the data were normally distributed; otherwise, the Kruskal–Wallis test was used. Statistical significance was set at *p*‐values < 0.05.

## Author Contributions

**Jin Jian**: Conceptualization, methodology, investigation, data curation, formal analysis, visualization, writing – original draft, funding acquisition. **Li Jia‐Jie**: conceptualization, methodology, investigation, data curation, visualization. **Tao Zi‐Han**: conceptualization, methodology, investigation, data curation. **Li Rong‐Jia**: investigation, formal analysis, writing – review and editing, funding acquisition. **Zhang Zi‐Liang**: methodology, investigation. **Liu Qing‐Song**: Methodology, writing – original draft. **Chen Zheng‐Li**: formal analysis, visualization. **Chen Ji‐Qiu**: investigation, visualization. **Wei Chen‐Ru**: methodology, data curation. **Liu Lei**: investigation, data curation. **Zhu Liang‐Liang**: conceptualization, supervision, writing – review and editing. **Zhu Shi‐Hui**: conceptualization, formal analysis, supervision, writing – review and editing, funding acquisition. **Lin Yun‐Feng**: conceptualization, supervision, writing – review and editing. All authors have read and approved the final manuscript.

## Ethics Statement

The animal experiments were approved by the Laboratory Animal Management and Use Committee of Huizhi Yinghua Medical Technology R&D (Shanghai) Co., Ltd. (approval number SH2022‐07024).

## Conflicts of Interest

Jian Jin, Rong‐Jia Li, and Zi‐Liang Zhang are employees in Shanghai Depeac Biotechnology Co., Ltd, but has no potential relevant financial or non‐financial interests to disclose. The other authors have no conflicts of interest to declare.

## Supporting information




**Figure S1**: tFNA concentration screening. (A) CCK8 assay showing the effect of different concentrations of tFNA on HaCaT proliferation. (B, C) EdU assay images (B) and quantification of HaCaT proliferation (C). (D, E) Scratch experiment images (D) and quantification of HaCaT migration (E). n = 5. *p < 0.05, **p < 0.01, ***p < 0.001. tFNA, tetrahedral framework nucleic acid; HaCaT, Human Immortalized Epidermal Cells; CCK8, cell counting kit‐8; EdU, 5‐ethynyl‐2'‐deoxyuridine.
**Figure S2**: tFNA uptake. (A) Flow cytometry plots showing tFNA uptake by HaCaT. (B) Immunofluorescence staining showing tFNA uptake by HSF and HMVEC. (C, D) Flow cytometry plots showing tFNA uptake by HSF (C) and HMVEC (D). n = 5. tFNA, tetrahedral framework nucleic acid; HaCaT, Human Immortalized Epidermal Cells; BJ, human skin fibroblasts; HMEC, human microvascular endothelial cells.
**Figure S3**: Biocompatibility of 100 nM tFNA. (A) Cytotoxicity, n = 10. (B) Intracutaneous stimulation, n = 3. (C) Allergenicity, n = 3. tFNA, tetrahedral framework nucleic acid.
**Figure S4**: Apoptosis and necrosis. (A, B) Immunofluorescence staining (A, green fluorescence for Caspase3) and quantification (B) of caspase 3 expression. (C, D). Immunofluorescence staining (C, green fluorescence for HMGB 1) and quantification (D) of HMGB1‐positive cells. n = 5. *p < 0.05, **p < 0.01, ***p < 0.001. HMGB1, high mobility group box 1.
**Figure S5**: Validation of gene knockdown. (A–J). Immunofluorescence staining (A) and quantification of LC3I (B), LC3II (C), LC3I/LC3II (D), P62 (E), mTOR (F), AMPK (G), p‐ULK1 (H), caspase 3 (I), and HMGB1 (J) expression. n = 5. *p < 0.05, **p < 0.01, ***p < 0.001. tFNA, tetrahedral framework nucleic acid; LC3I, low complexity communications codec I; LC3II, low complexity communications codec II; P62, ubiquitin‐binding protein p62; mTOR, mammalian target of rapamycin; AMPK, adenosine 5′‐monophosphate‐activated protein kinase; p‐ULK1, phosphorylated Unc‐51‐like autophagy protein 1; HMGB1, high mobility group box 1.
**Figure S6**: tFNA concentration screening in animal experiments. (A) General photo. (B) Healing rate of 9 D. (C) Healing rate of 18 D. (D) Healing time. n = 6. *p < 0.05, **p < 0.01, ***p < 0.001. tFNA, tetrahedral framework nucleic acid.
**Figure S7**: Apoptosis, necrosis, and angiogenesis in wound tissue. (A, B) Immunofluorescence staining (A) and quantification (B) of caspase 3 expression as a marker of apoptosis. (C, D) Immunofluorescence staining (C) and quantification (D) of HMGB1 expression as a marker of necrosis. (E, F) Immunofluorescence staining (E) and quantification (F) of CD31 expression as a marker of angiogenesis. n = 15. *p < 0.05, **p < 0.01, ***p < 0.001. HMGB1, high mobility group box 1; CD31, Platelet endothelial cell adhesion molecule‐1.

## Data Availability

The raw/processed data are available from the corresponding authors upon reasonable request.

## References

[1] J. Jian , P. Yu , C. Zhengli , et al., “Determining Transfusion Use in Major Burn Patients: A Retrospective Review and Analysis From 2009 to 2019,” Burns 48, no. 5 (2022): 1104–1111. 34839960 10.1016/j.burns.2021.09.004

[2] Y. Bordon , “Hypoxia and IL‐24 Drive a Sterile Wound Healing Pathway,” Nature Reviews Immunology 23, no. 6 (2023): 344. 10.1038/s41577-023-00888-4 PMC10171156 37165170

[3] K. M. Sullivan , H. P. Lorenz , M. Meuli , R. Y. Lin , and N. S. Adzick , “A Model of Scarless human Fetal Wound Repair Is Deficient in Transforming Growth Factor Beta,” Journal of Pediatric Surgery 30, no. 2 (1995): 198–202; discussion 202–203. 7738738 10.1016/0022-3468(95)90560-x

[4] J. Jin , T. Tang , H. Zhou , et al., “Synergistic Effects of Quercetin‐modified Silicone Gel Sheet in Scar Treatment,” Journal of Burn Care & Research 43, no. 2 (2022): 445–452. 34089615 10.1093/jbcr/irab100

[5] R. Luo , Y. Liang , J. Yang , et al., “Reshaping the Endogenous Electric Field to Boost Wound Repair via Electrogenerative Dressing,” Advanced Materials 35, no. 16 (2023): e2208395. 36681867 10.1002/adma.202208395

[6] D. Zhao , D. Xiao , M. Liu , et al., “Tetrahedral Framework Nucleic Acid Carrying Angiogenic Peptide Prevents Bisphosphonate‐Related Osteonecrosis of the Jaw by Promoting Angiogenesis,” International Journal of Oral Science 14, no. 1 (2022): 23. 35477924 10.1038/s41368-022-00171-7 PMC9046247

[7] I. Bártolo , R. L. Reis , A. P. Marques , and M. T. Cerqueira , “Keratinocyte Growth Factor‐Based Strategies for Wound Re‐Epithelialization,” Tissue Engineering, Part B: Reviews 28, no. 3 (2022): 665–676. 34238035 10.1089/ten.TEB.2021.0030

[8] C. Zhou , D. Guan , J. Guo , et al., “Correction: Human Parathyroid Hormone Analog (3–34/29–34) Promotes Wound Re‐Epithelialization Through Inducing Keratinocyte Migration and Epithelial–Mesenchymal Transition via PTHR1‐PI3K/AKT Activation,” Cell Communication and Signaling 21, no. 1 (2023): 243. 37726814 10.1186/s12964-023-01318-7 PMC10507821

[9] M. Migliario , P. Yerra , S. Gino , M. Sabbatini , and F. Renò , “Laser Biostimulation Induces Wound Healing‐Promoter β2‐Defensin Expression in Human Keratinocytes via Oxidative Stress,” Antioxidants 12, no. 8 (2023): 2076–3921. 37627545 10.3390/antiox12081550 PMC10451672

[10] M. Zhou , Y. Lu , Y. Tang , et al., “A DNA‐Based Nanorobot for Targeting, Hitchhiking, and Regulating Neutrophils to Enhance Sepsis Therapy,” Biomaterials 318 (2025): 123183. 39951831 10.1016/j.biomaterials.2025.123183

[11] T. Zhang , H. Ma , X. Zhang , S. Shi , and Y. Lin , “Functionalized DNA Nanomaterials Targeting Toll‐Like Receptor 4 Prevent Bisphosphonate‐Related Osteonecrosis of the Jaw via Regulating Mitochondrial Homeostasis in Macrophages,” Advanced Functional Materials 33, no. 15 (2023): 2213401.

[12] M. Zhou , Y. Tang , Y. Lu , et al., “Framework Nucleic Acid‐Based and Neutrophil‐Based Nanoplatform Loading Baicalin With Targeted Drug Delivery for Anti‐Inflammation Treatment,” ACS Nano 19, no. 3 (2025): 3455–3469. 39817852 10.1021/acsnano.4c12917

[13] S. Li , Y. Liu , T. Zhang , et al., “A Tetrahedral Framework DNA‐Based Bioswitchable miRNA Inhibitor Delivery System: Application to Skin Anti‐Aging,” Advanced Materials 34, no. 46 (2022): e2204287. 35901292 10.1002/adma.202204287

[14] D. Mijaljica , F. Spada , D. J. Klionsky , and I. P. Harrison , “Autophagy Is the Key to Making Chronic Wounds Acute in Skin Wound Healing,” Autophagy 19, no. 9 (2023): 2578–2584. 36994997 10.1080/15548627.2023.2194155 PMC10392758

[15] J. Jin , K. S. Zhu , S. M. Tang , et al., “Trehalose Promotes Functional Recovery of Keratinocytes Under Oxidative Stress and Wound Healing via ATG5/ATG7,” Burns 49, no. 6 (2023): 1382–1391. 36759218 10.1016/j.burns.2022.11.014

[16] E. Caves and V. Horsley , “Reindeer Light the Way to Scarless Wound Healing,” Cell 185, no. 25 (2022): 4675–4677. 36493748 10.1016/j.cell.2022.11.013

[17] T. Tian , Y. Li , and Y. Lin , “Prospects and Challenges of Dynamic DNA Nanostructures in Biomedical Applications,” Bone Research 10, no. 1 (2022): 40. 35606345 10.1038/s41413-022-00212-1 PMC9125017

[18] T. Zhang , T. Tian , and Y. Lin , “Functionalizing Framework Nucleic‐Acid‐Based Nanostructures for Biomedical Application,” Advanced Materials 34, no. 46 (2022): e2107820. 34787933 10.1002/adma.202107820

[19] J. Karregat , T. Rustemeyer , and S. A. S. van der Bent , “Assessment of Cytotoxicity and Sensitization Potential of Intradermally Injected Tattoo Inks in Reconstructed Human Skin,” Contact Dermatitis 85, no. 3 (2021): 324–339. 34029376 10.1111/cod.13908 PMC8453820

[20] R. El‐Khoury , M. Rak , P. Bénit , H. T. Jacobs , and P. Rustin , “Cyanide Resistant Respiration and the Alternative Oxidase Pathway: A Journey From Plants to Mammals,” Biochimica et Biophysica (BBA) – Bioenergetics 1863, no. 6 (2022): 148567. 10.1016/j.bbabio.2022.148567 35500614

[21] Y. Liu , S. Li , S. Wang , et al., “LIMP‐2 Enhances Cancer Stem‐Like Cell Properties by Promoting Autophagy‐Induced GSK3β Degradation in Head and Neck Squamous Cell Carcinoma,” International Journal of Oral Science 15, no. 1 (2023): 24. 37291150 10.1038/s41368-023-00229-0 PMC10250453

[22] X. Huang , J. Yao , L. Liu , et al., “S‐acylation of p62 Promotes p62 Droplet Recruitment Into Autophagosomes in Mammalian Autophagy,” Molecular Cell 83, no. 19 (2023): 3485–3501.e11. 37802024 10.1016/j.molcel.2023.09.004 PMC10552648

[23] X. Ning , K. Yang , W. Shi , and C. Xu , “Comparison of Hypertrophic Scarring on a Red Duroc Pig and a Guangxi Mini Bama Pig,” Scars, Burns & Healing 6 (2020): 2059513120930903. 10.1177/2059513120930903 PMC7318807 32637158

[24] Y. Liu , J. Y. Chen , H. T. Shang , et al., “Light Microscopic, Electron Microscopic, and Immunohistochemical Comparison of Bama Minipig (*Sus scrofa domestica*) and Human Skin,” Comparative Medicine 60, no. 2 (2010): 142–148. 20412690 PMC2855042

[25] R. Chen , D. Wen , W. Fu , et al., “Treatment Effect of DNA Framework Nucleic Acids on Diffuse Microvascular Endothelial Cell Injury After Subarachnoid Hemorrhage,” Cell Proliferation 55, no. 4 (2022): e13206. 35187748 10.1111/cpr.13206 PMC9055902

[26] US Food and Drug Administration . ISO 10993‐12:2012: Biological Evaluation of Medical Devices — Part 12: Sample Preparation and Reference Materials. 4th ed. (ISO, 2012).

[27] Y. Wang , C. He , C. Chen , et al., “Thermoresponsive Self‐Healing Zwitterionic Hydrogel as an In Situ Gelling Wound Dressing for Rapid Wound Healing,” ACS Applied Materials & Interfaces 14, no. 50 (2022): 55342–55353. 36473731 10.1021/acsami.2c15820

[28] J. Ai , H. Wan , M. Shu , et al., “Guhong Injection Protects Against Focal Cerebral Ischemia–Reperfusion Injury via Anti‐Inflammatory Effects in Rats,” Archives of Pharmacal Research 40, no. 5 (2017): 610–622. 27624481 10.1007/s12272-016-0835-4

[29] Y. Song , Y. Cheng , T. Lan , et al., “ERK Inhibitor: A Candidate Enhancing Therapeutic Effects of Conventional Chemo‐Radiotherapy in Esophageal Squamous Cell Carcinoma,” Cancer Letters 554 (2023): 216012. 36470544 10.1016/j.canlet.2022.216012

[30] X. Lyu , F. Cui , H. Zhou , et al., “3D Co‐culture of Macrophages and Fibroblasts in a Sessile Drop Array for Unveiling the Role of Macrophages in Skin Wound‐Healing,” Biosensors & Bioelectronics 225 (2023): 115111. 36731395 10.1016/j.bios.2023.115111

[31] J. Jin , X. F. Zheng , F. He , et al., “Therapeutic Efficacy of Early Photobiomodulation Therapy on the Zones of Stasis in Burns: An Experimental Rat Model Study,” Wound Repair and Regeneration 26, no. 6 (2018): 426–436. 30118166 10.1111/wrr.12661

