# Correction to “Keratinocyte Autophagy‐Mediated Self‐Assembling Tetrahedral Framework Nucleic Acid Induces Wound Healing and Reduces Scar Hyperplasia”

**DOI:** 10.1002/mco2.70578

**Published:** 2026-01-07

Jian Jin, Jiajie Li, Zihan Tao, et al., *Keratinocyte autophagy‐mediated self‐assembling tetrahedral framework nucleic acid induces wound healing and reduces scar hyperplasia*. MedComm 6 (2025): e70355.

1. The immunofluorescence image of the ATG5 + ATG7 experimental group for 2 h in Figure 3A is incorrect and has been changed to the following image:


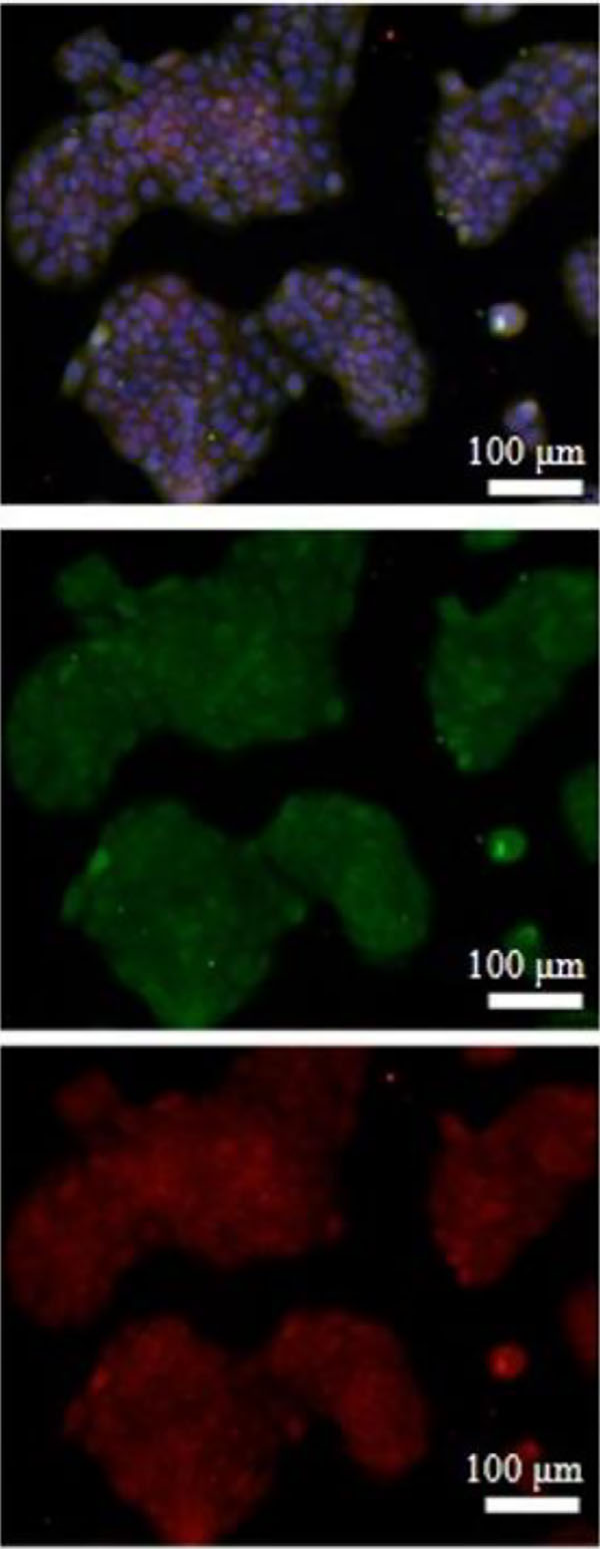


2. The image size of the experimental group in Figure 5A after 90 days of healing is incorrect and has been changed to the following image:


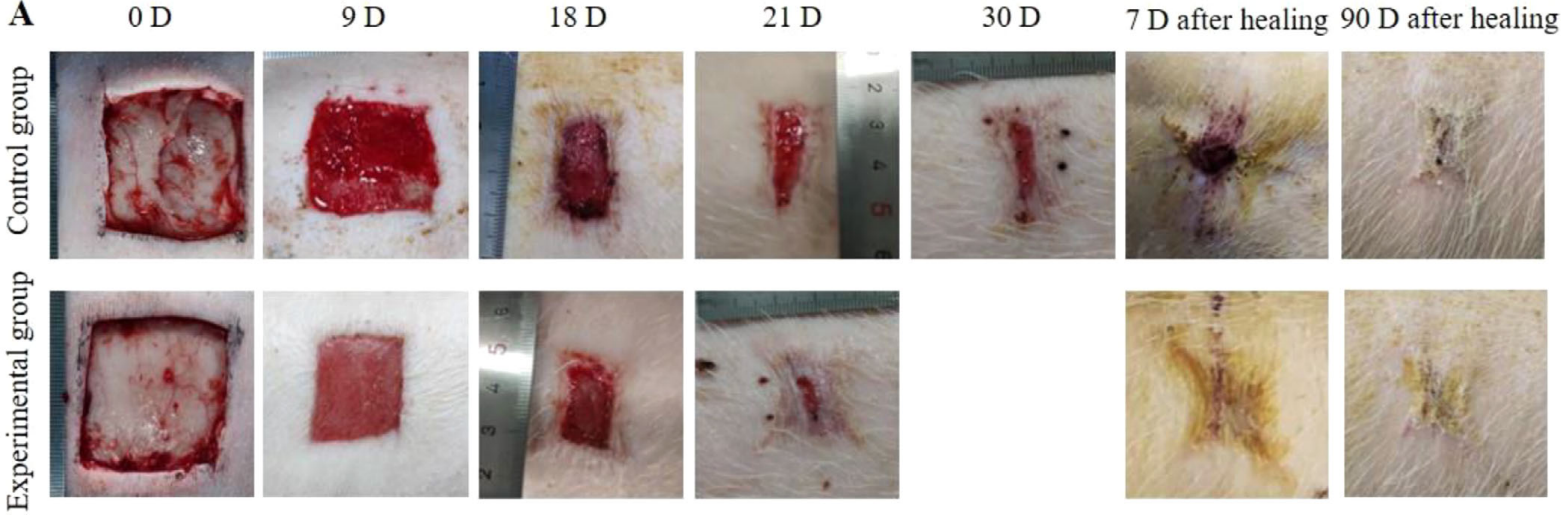


3. The 50 nM in Figure S1D is incorrect and has been changed to the following figure:


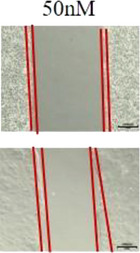


4. The 0 day image of 150 nM in Figure S6A is incorrect and has been changed to the following image:


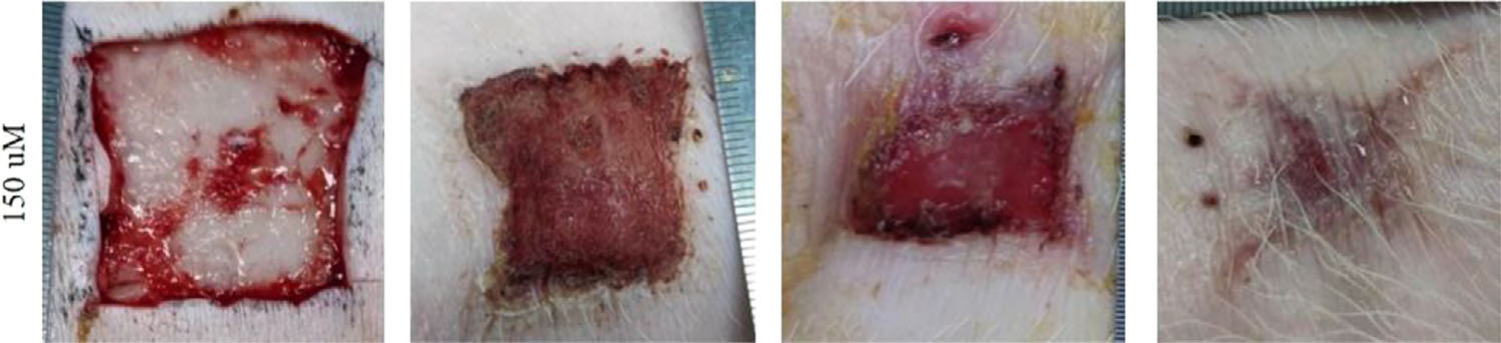


The authors apologize for these errors.

