# Supplementary material for: Keratinocyte Autophagy‐Mediated Self‐Assembling Tetrahedral Framework Nucleic Acid Induces Wound Healing and Reduces Scar Hyperplasia
Source: MedComm (2020). 2025 Sep 16;6(10):e70355. doi: 10.1002/mco2.70355 (PMC12441305; doi:10.1002/mco2.70355)
Supplement: Supplementary file 1 — Figure S1: tFNA concentration screening. (A) CCK8 assay showing the effect of different concentrations of tFNA on HaCaT proliferation. (B, C) EdU assay images (B) and quantification of HaCaT proliferation (C). (D, E) Scratch experiment images (D) and quantification of HaCaT migration (E). n = 5. *p < 0.05, **p < 0.01, ***p < 0.001. tFNA, tetrahedral framework nucleic acid; HaCaT, Human Immortalized Epidermal Cells; CCK8, cell counting kit‐8; EdU, 5‐ethynyl‐2'‐deoxyuridine. Figure S2: tFNA uptake. (A) Flow cytometry plots showing tFNA uptake by HaCaT. (B) Immunofluorescence staining showing tFNA uptake by HSF and HMVEC. (C, D) Flow cytometry plots showing tFNA uptake by HSF (C) and HMVEC (D). n = 5. tFNA, tetrahedral framework nucleic acid; HaCaT, Human Immortalized Epidermal Cells; BJ, human skin fibroblasts; HMEC, human microvascular endothelial cells. Figure S3: Biocompatibility of 100 nM tFNA. (A) Cytotoxicity, n = 10. (B) Intracutaneous stimulation, n = 3. (C) Allergenicity, n = 3. tFNA, tetrahedral framework nucleic acid. Figure S4: Apoptosis and necrosis. (A, B) Immunofluorescence staining (A, green fluorescence for Caspase3) and quantification (B) of caspase 3 expression. (C, D). Immunofluorescence staining (C, green fluorescence for HMGB 1) and quantification (D) of HMGB1‐positive cells. n = 5. *p < 0.05, **p < 0.01, ***p < 0.001. HMGB1, high mobility group box 1. Figure S5: Validation of gene knockdown. (A–J). Immunofluorescence staining (A) and quantification of LC3I (B), LC3II (C), LC3I/LC3II (D), P62 (E), mTOR (F), AMPK (G), p‐ULK1 (H), caspase 3 (I), and HMGB1 (J) expression. n = 5. *p < 0.05, **p < 0.01, ***p < 0.001. tFNA, tetrahedral framework nucleic acid; LC3I, low complexity communications codec I; LC3II, low complexity communications codec II; P62, ubiquitin‐binding protein p62; mTOR, mammalian target of rapamycin; AMPK, adenosine 5′‐monophosphate‐activated protein kinase; p‐ULK1, phosphorylated Unc‐51‐like autophagy protein 1; HMGB1, high mobili [file MCO2-6-e70355-s001.docx]

**Keratinocyte autophagy-mediated self-assembling tetrahedral framework nucleic acid (tFNA) induces wound healing and reduces scar hyperplasia**

**Supporting Information**

**Supporting Methods**

**Vancouver scar scale**

Melanin:

0 points, scar color is similar to the skin color of normal parts of the body

1 point, light color

2 points, mixed color

3 points, with a darker color

Vascularity:

0 points, scar skin color is similar to normal body parts

1 point, with a pinkish skin tone

2 points, with a reddish skin tone

3 points, with purple skin color

Pliability:

0 points, normal

1 point, soft (able to deform the skin with minimal resistance)

2 points, compliant (able to deform under pressure)

3 points, hard (unable to deform, moving in block shape, resistant to pressure)

4 points, curved (tissue like rope, scar will retract when stretched)

5 points, contracture (permanent shortening of scars leading to disability and distortion)

Height:

0 minutes, normal

1 point, <1 mm

2 points, 1–2 mm

3 points, 2–4 mm

4 points, >4 mm

**Supporting Figures**


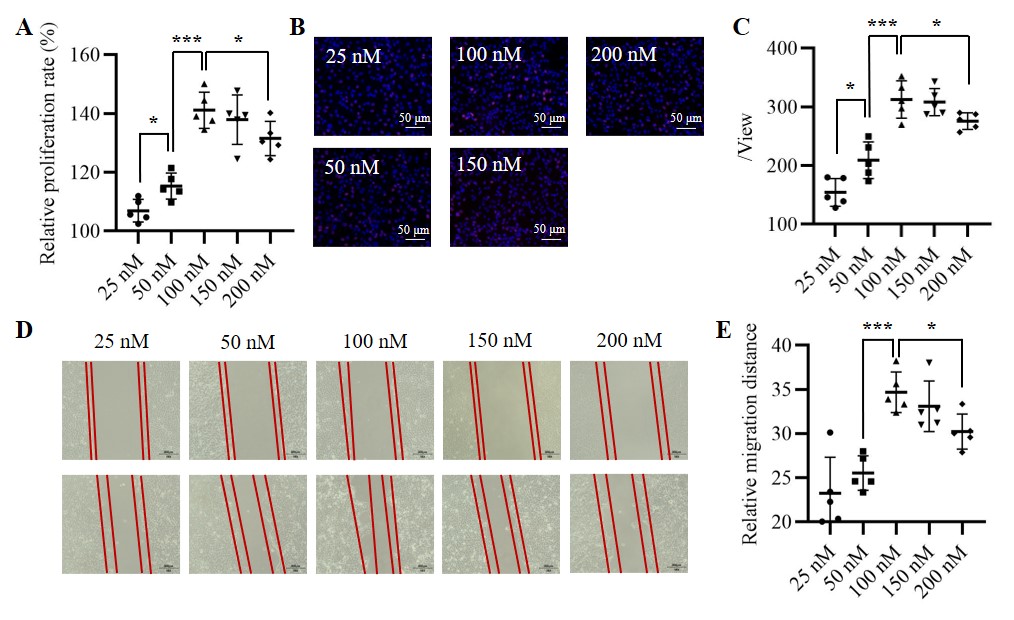


**Figure S1.** tFNA concentration screening. (A) CCK8 assay showing the effect of different concentrations of tFNA on HaCaT proliferation. (B, C) EdU assay images (B) and quantification of HaCaT proliferation (C). (D, E) Scratch experiment images (D) and quantification of HaCaT migration (E). n=5. *p < 0.05, **p < 0.01, ***p < 0.001. tFNA, tetrahedral framework nucleic acid; HaCaT, [Human Immortalized Epidermal Cells](https://www.baidu.com/s?wd=Human%20Immortalized%20Epidermal%20Cells&rsv_idx=2&tn=baiduhome_pg&usm=2&ie=utf-8&rsv_pq=a5517edc001de281&oq=HaCaT%E5%85%A8%E7%A7%B0&rsv_t=949d179B9p2aM2H5U1UVbSsVk0hNpbBbvieajSxHXhputqrgvSQCfXCSufG1N3gn7mMf&rsv_dl=re_dqa_generate&sa=re_dqa_generate); CCK8, [cell counting kit-8](https://www.baidu.com/baidu.php?url=Kf0000K5cNxA6dzipyi9w4Cj5mduJ1r7JPiRlHdtAVWF0twMu9bSNnBful58zS5NFg_PQDvjP7nhwdmHJF3vwn8uTBOp6FXNm3cJ0cSUIc6r1ujda-9ug7zitgi5qyAAtSCZx-QfKkPLeud8JyhJ6Ny51vpkV7UkhPlxe4pECi2l9wnS-IpULYDTMquF6M_DMctgrYeRf-4xPMTTpf9ndQ5t7fMM.7D_KMAMCEXeb69HwC43gmcJjtvxVxf1entn5UMvTdPHV2XgZJyAp7WIvIr1GR0.U1Yz0ZDqfYPQr0KspynqnfKY5yPWpzRznj6-nWjRoTjV0A-V5HczPfKM5yq-TZns0ZNG5yF9pywd0ZKGujYz0APGujYYnjm0UgfqnH0krNtknjDLg1csPH7xn1msnfKopHYs0ZFY5HRsP0KBpHYkPH9xnW0Y0AdW5HDsnH-xnH0kPdtznjRkg100TgKGujYs0Z7Wpyfqn0KzuLw9u1Ys0A49pyfqn0K8mgws5H00mLNb5HRLnWbvPWf40A7B5HKxn0K-ThTqn0KsTjYkPjDYP1b1rjRz0A4vTjYsQW0snj0snj0s0AdYTjYs0AwbUL0qn0KzpWYs0Aw-IWdsmsKhIjYs0ZKC5H00ULnqn0KBI1Ykn0K8IjYs0ZPl5fK9TdqGuAnqTZP9ThYVuyPJ0A-bm1dri6KspZw45fKYmgFMugfqn17xn1DYg1DknfKYIgnqPWcdP1mYnjD4PHb1PWfYrHRzPfKzug7Y5HDLPjT1PWT3rjf1njb0pynqn0K1pyfqrymznWf1rHcsnjDsryc1n6KWTvYqrjTLnWF7nbn3PjPjrHwDP6K_mMnqn0K9m1Yk0ZK85H00TydY5H00Tyd15H00XMfqn0KVmdqhThqV5HKxn7tsg100uA78IyF-gLK_my4GuZnqn7tsg1Kxn0Ksmgwxuhk9u1Ys0AwWpyfqn0K-IA-b5iYk0A71TAPW5H00IgKGUhPW5H00Tydh5H00uhPdIjYs0A-1mvsqn0K9uAu_myTqnfK_uhnqn0KbmvPb5H0znD7Dwbf4njmYnWRkfHDsrjDzPbcknRNjwWRzwWn1x7uQid7Ni1Fuy0KYTh7buHYvn10znjm0mhwGujY4fWu7rDuAwRuawbD4rj0snHIjfW0LnWNDnWIafWuaf6KEm1Yk0AFY5HD0Uv7YI1Ys0AqY5Hc0ULFsIjYzc10WPH_4nBnknj0sc1DvnHT1P1R4n1cWnansQW0snj0snankc1Dsnj0WnanVc108njcsnj0sc1D8nj0snj0sc10WnansQW0snj0snansc10Wnansc10Wnans0AF9UhV9mvnqnansc10Wn0K3TLwd5HD3PHbvn1c40Z7xIWYsQW6dg108njKxna3sn7tsQW6dg108PH9xn7tsQWR3g100mMPxTZFEuA-b5H00ThqGuhk9u1Ys0APv5fKGTdqWTADqn0KWTjYs0AN1IjYs0Z7MIvfqn0KETjDqn0KsTjfqn6KWThnqPHb1njT&us=newvui&xst=TjYkPjDYP1b1rjRz0ynqnjcsfRwAwjbsPWfzPH7KnH03nHcvfWDkwRPAPHFAn1PtNbVQRNNQn--m0ycqrRcvwH9AwbNAfbuKrH6snjDLfYcsP1cdwjcLfbcvfbcKT1YknHfvPW6snH64PHR3Pj0LrjmYn-tznWNxnf7L5yPWpz03cPiVsrYKTHdWmv_30gRqnH6drHm1nWbKIjYkP1fLn1mLrj6Y0ydk5H0an0cV0yPC5yuWgLKW0ykd5H0Kmv3qmh7GuZNCUvd-gLKM0gFY5H0Kn1fYPWmkn1TYPf&cegduid=nH6drHm1nWb&solutionId=5766571&word=&ck=6969.5.3515.0.0.419.219.0&shh=www.baidu.com&sht=baiduhome_pg&wd=); EdU, 5-ethynyl-2'-deoxyuridine.


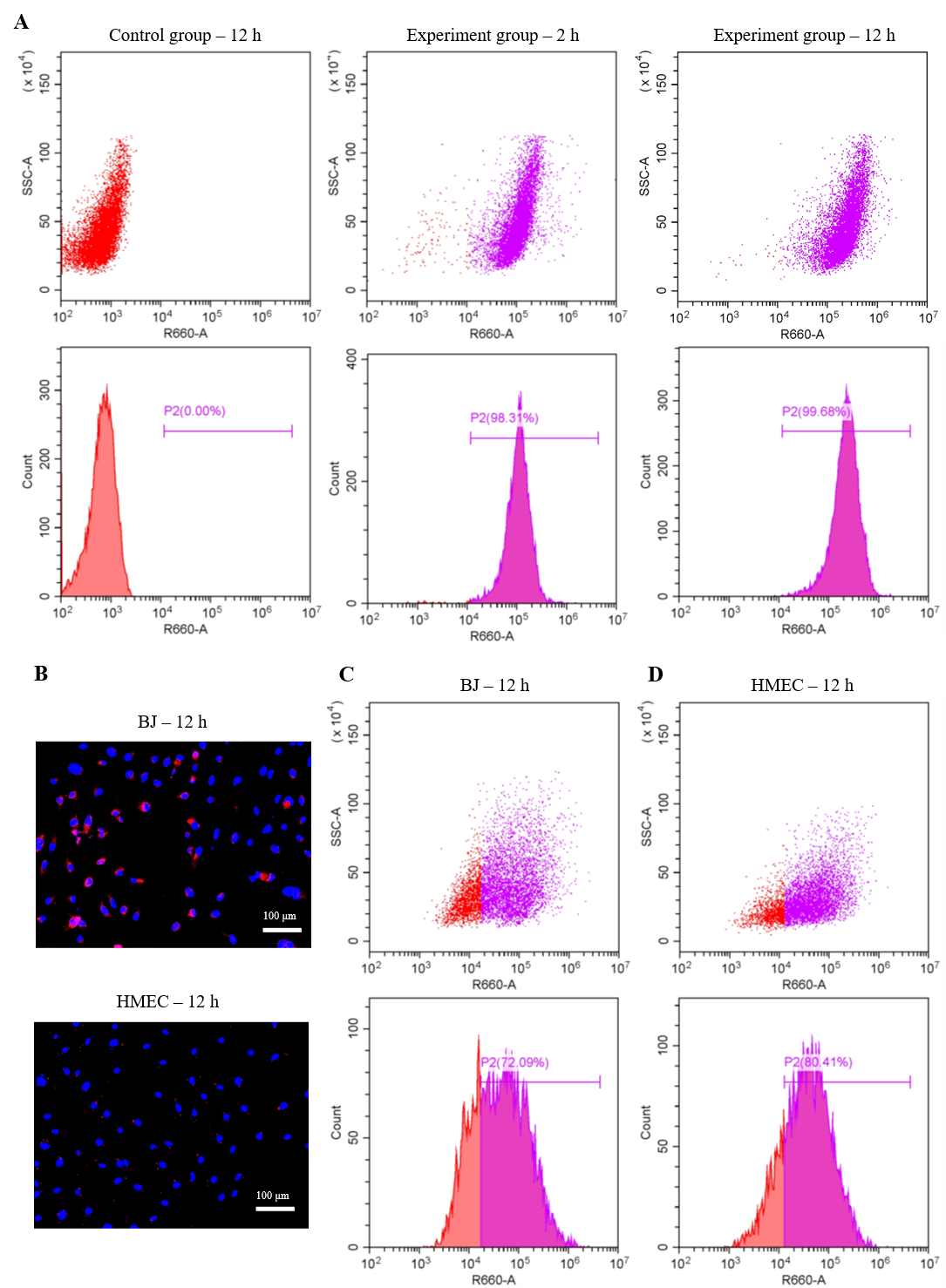


**Figure S2.** tFNA uptake. (A) Flow cytometry plots showing tFNA uptake by HaCaT. (B) Immunofluorescence staining showing tFNA uptake by HSF and HMVEC. (C, D) Flow cytometry plots showing tFNA uptake by HSF (C) and HMVEC (D). n=5. tFNA, tetrahedral framework nucleic acid; HaCaT, [Human Immortalized Epidermal Cells](https://www.baidu.com/s?wd=Human%20Immortalized%20Epidermal%20Cells&rsv_idx=2&tn=baiduhome_pg&usm=2&ie=utf-8&rsv_pq=a5517edc001de281&oq=HaCaT%E5%85%A8%E7%A7%B0&rsv_t=949d179B9p2aM2H5U1UVbSsVk0hNpbBbvieajSxHXhputqrgvSQCfXCSufG1N3gn7mMf&rsv_dl=re_dqa_generate&sa=re_dqa_generate); BJ, human skin fibroblasts ; HMEC, human microvascular endothelial cells.


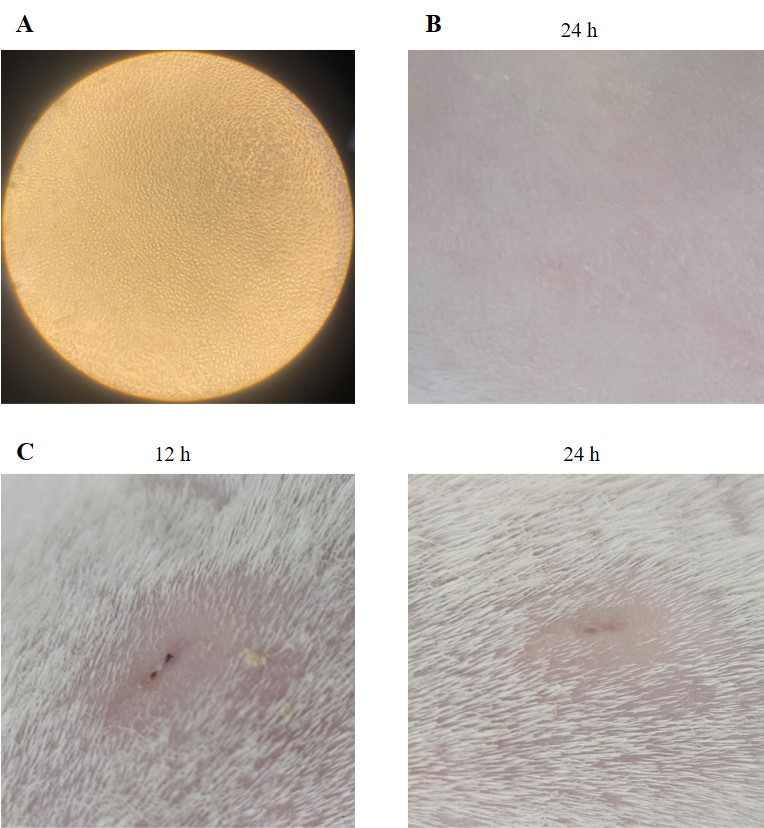


**Figure S3.** Biocompatibility of 100 nM tFNA. (A) Cytotoxicity, n=10. (B) Intracutaneous stimulation, n=3. (C) Allergenicity, n=3. tFNA, tetrahedral framework nucleic acid.


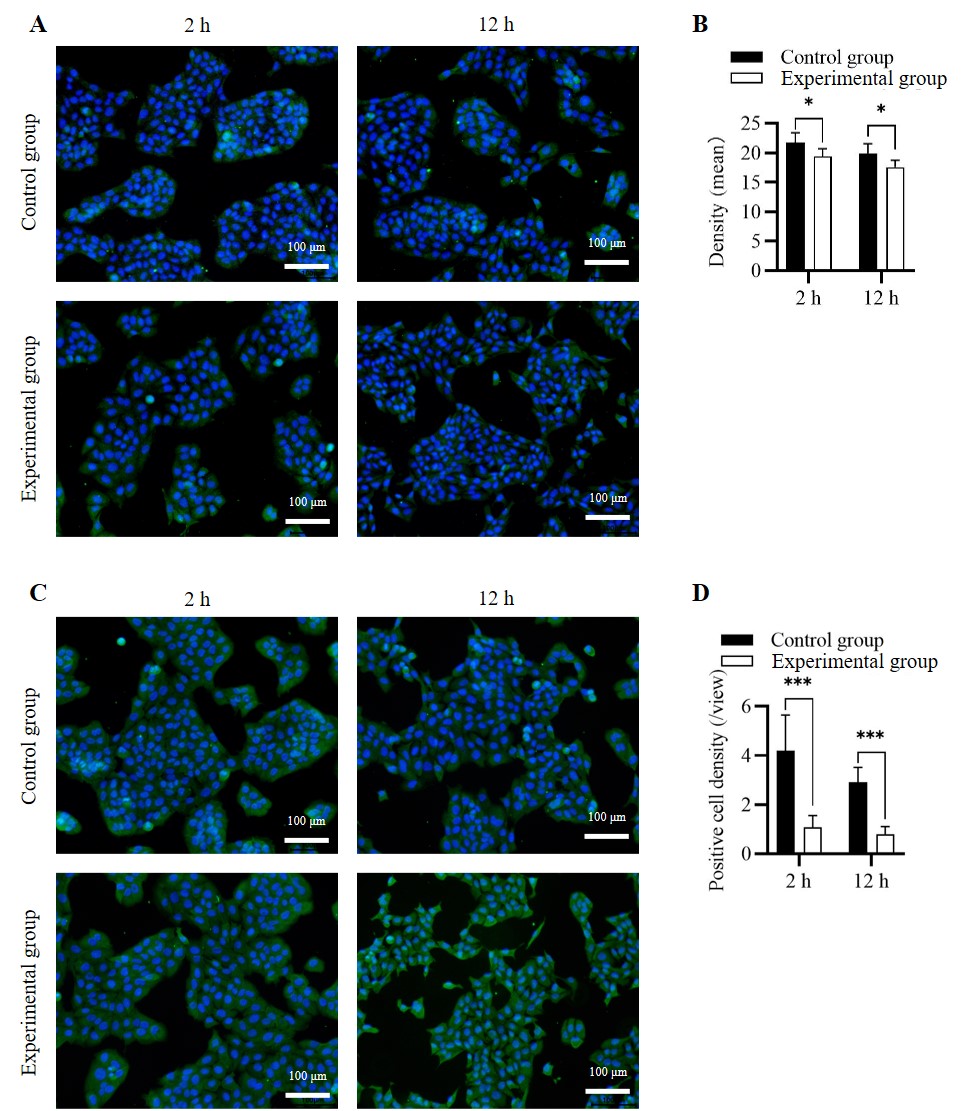


**Figure S4.** Apoptosis and necrosis. (A, B) Immunofluorescence staining (A, green fluorescence for Caspase3) and quantification (B) of caspase 3 expression. (C, D). Immunofluorescence staining (C, green fluorescence for HMGB 1) and quantification (D) of HMGB1-positive cells. n=5. *p < 0.05, **p < 0.01, ***p < 0.001. HMGB1, high mobility group box 1.

**
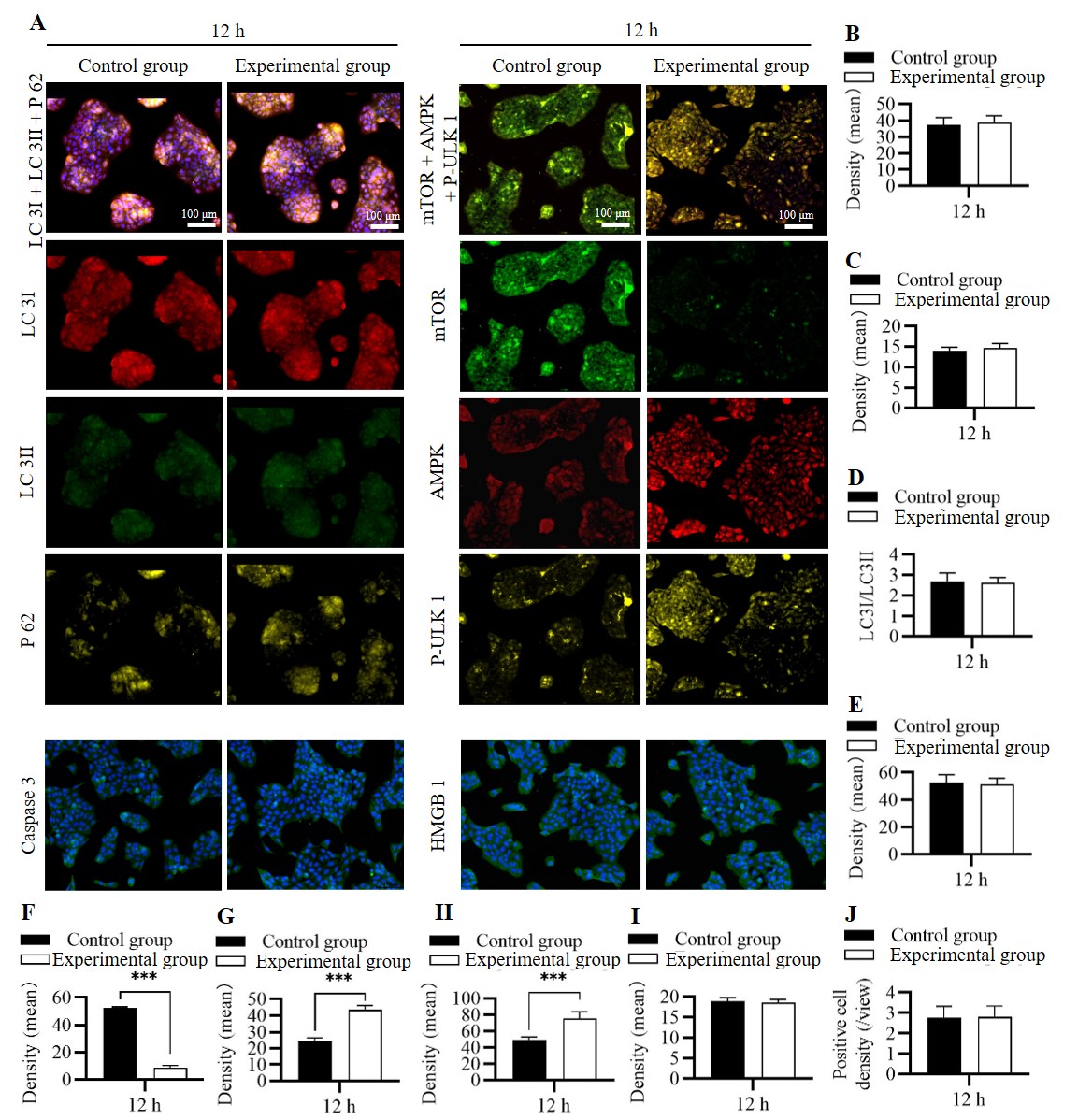
**

**Figure S5.** Validation of gene knockdown. (A–J). Immunofluorescence staining (A) and quantification of LC3I (B), LC3II (C), LC3I/LC3II (D), P62 (E), mTOR (F), AMPK (G), p-ULK1 (H), caspase 3 (I), and HMGB1 (J) expression. n=5. *p < 0.05, **p < 0.01, ***p < 0.001. tFNA, tetrahedral framework nucleic acid; LC3I, low complexity communications codec I; LC3II, low complexity communications codec II; P62, ubiquitin-binding protein p62; mTOR, mammalian target of rapamycin; AMPK, adenosine 5′-monophosphate-activated protein kinase; p-ULK1, phosphorylated Unc-51-like autophagy protein 1; HMGB1, high mobility group box 1.


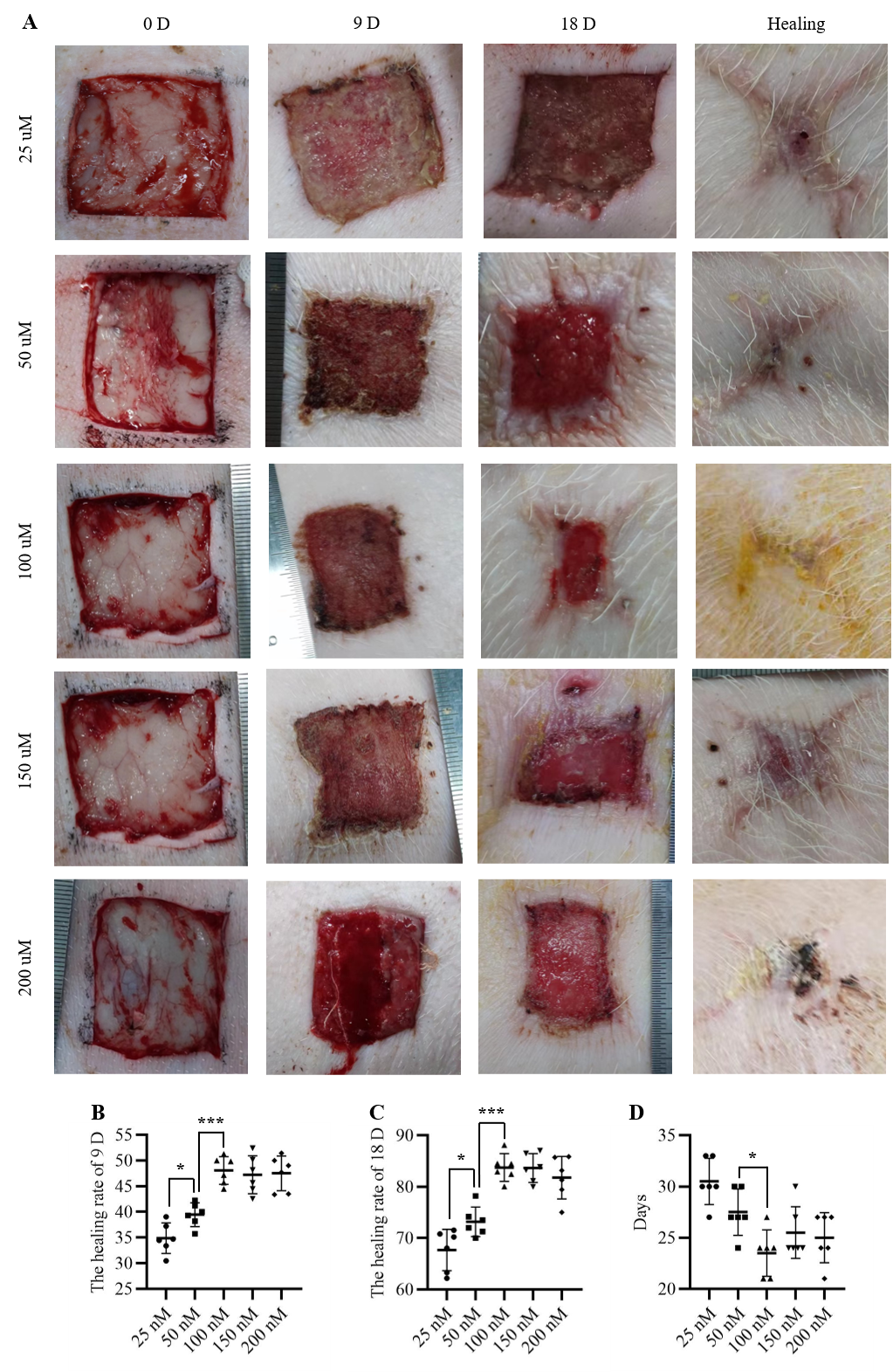
 **Figure S6.** tFNA concentration screening in animal experiments. (A) General photo. (B) Healing rate of 9 D. (C) Healing rate of 18 D. (D) Healing time. n=6. *p < 0.05, **p < 0.01, ***p < 0.001. tFNA, tetrahedral framework nucleic acid.


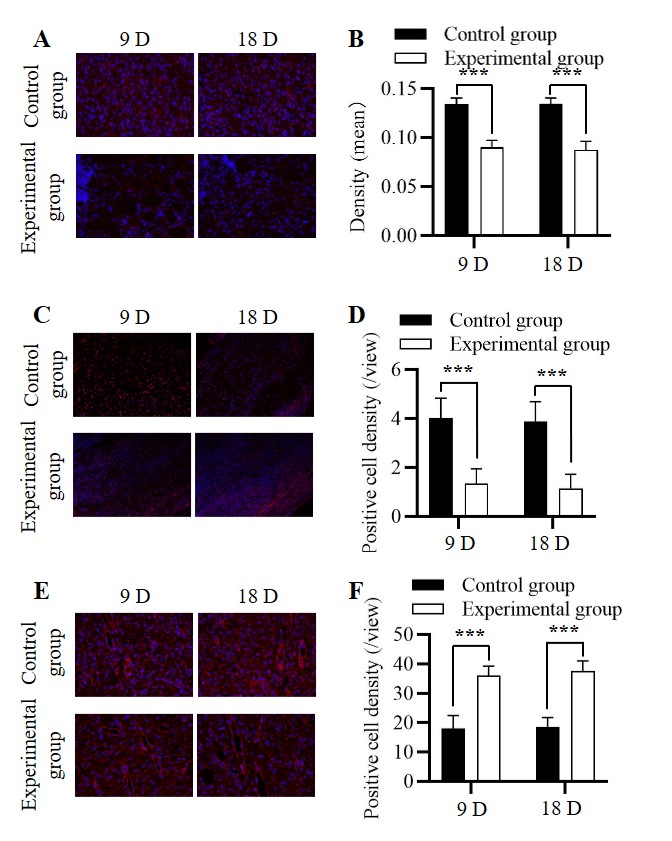


**Figure S7.** Apoptosis, necrosis, and angiogenesis in wound tissue. (A, B) Immunofluorescence staining (A) and quantification (B) of caspase 3 expression as a marker of apoptosis. (C, D) Immunofluorescence staining (C) and quantification (D) of HMGB1 expression as a marker of necrosis. (E, F) Immunofluorescence staining (E) and quantification (F) of CD31 expression as a marker of angiogenesis. n=15. *p < 0.05, **p < 0.01, ***p < 0.001. HMGB1, high mobility group box 1; CD31, Platelet endothelial cell adhesion molecule-1.
